# Unraveling site-specific seed formation abnormalities in *Picea neoveitchii* Mast. trees via widely metabolomic and transcriptomic analysis

**DOI:** 10.3389/fpls.2024.1495784

**Published:** 2024-12-10

**Authors:** Kaiyuan Li, Jiayi Lin, Rong Fan, Sibo Chen, Zhilin Ma, Wenli Ji

**Affiliations:** ^1^ College of Landscape Architecture and Art, Northwest A&F University, Yangling, China; ^2^ Institute of Wetland and Grassland Conservation, Shaanxi Academy of Forestry, Xian, China

**Keywords:** conifer, empty seed shell, endogenous hormones, sugar, widely metabolomic, transcriptomic analysis

## Abstract

*Picea neoveitchii* Mast. is a rare and threatened species of evergreen coniferous tree in China, commonly facing issues such as damaged seeds, abnormal seed growth, and empty seed shells. These abnormalities vary by location; unfortunately, the reasons behind these inconsistencies are completely unknown. This study compared seeds from two 150-year-old trees located in Taibai (Shaanxi province, TB150) and Zhouqu (Gansu province, ZQ150). The results showed significant differences in 43 metabolites and hormone levels, with higher levels of indole-3-acetic acid (IAA), methyl jasmonate (MeJA), and brassinosteroid (BR) in ZQ150, which were associated with more viable seeds. In contrast, TB150 exhibited more damaged seeds and empty seed shells due to higher abscisic acid (ABA) levels. Moreover, to further investigate these inconsistencies, we performed *de-novo* transcriptomic assembly and functional annotation of unigenes using high-throughput sequencing. A total of 2,355 differentially expressed unigenes were identified between TB150 and ZQ150, with 1,280 upregulated and 1,075 downregulated. Hormone signaling and sugar metabolism-related unigenes were further examined for their role in seed development. ZQ150 increased the number of normal seeds by enhancing endogenous IAA levels and upregulating auxin signaling and sugar metabolism-related genes. Conversely, TB150 showed more empty seed shells, correlated with elevated ABA levels and the activation of ABA signaling genes. We hypothesize that enhanced IAA levels and the upregulation of sugar metabolism and auxin signaling genes promote normal seed development.

## 1 Introduction

*Picea neoveitchii* Mast. is a conifer species in the Pinaceae family, found only in China. Conifers have male and female cones, which each generates an ovule and pollen. A pollen grain fertilizes an ovule, which then produces seed. The ovuliferous scales and bracts on the female cones, containing the seed, are typically organized spirally around a central axis. In the majority of species, each ovuliferous scale along the cone axis has two ovules, each of which is capable of developing a viable seed. The proportion of scales that can generate viable seeds differs by species and may not develop seeds at the base or tip of the cone (Kolotelo, 1997). The propagation of *P. neoveitchii* essentially depends on seeds. However, extremely empty seed shells and undeveloped seed growth result in substantial losses in yield and propagation. The intensity of empty seed shells varies at different locations within China. Empty seed shells can result from a multifaceted interplay of factors, such as physiological, molecular, genetic, sugar contents, pests and diseases, and environmental aspects. Unfortunately, studies on the underlying mechanisms regulating seed formation or empty seed shells in *P. neoveitchii* remain unclear.

Among endogenous hormones, auxins have significance in several stages of seed growth, including ovule formation, which is the precursor to seed formation. Indole-3-acetic acid (IAA) aids in the initiation of embryonic growth and is essential for seed formation and development (Friml, 2003; Moubayidin et al., 2010). A lack of auxin or an imbalance in their distribution inside the tree can cause inconsistencies in seed development, perhaps resulting in empty seed shells. Abscisic acid (ABA) mainly plays a role in seed maturation, dormancy, and stress tolerance (Rodríguez-Gacio et al., 2009). However, excessive ABA concentration or distribution in its signaling pathways can culminate in seed development defects such as seed abortion or the generation of non-viable seeds (Cutler et al., 2010). Gibberellins (GAs) also play a role in seed germination and embryonic growth. During seed development, they promote cell elongation and expansion (Yamaguchi, 2008). It is well known that cytokinins (CK) control cell division and differentiation during seed formation, which helps in embryonic development (Riefler et al., 2006). Other hormones, such as brassinosteroid (BR) and methyl jasmonate (MeJA), may play key roles in seed formation. Unfortunately, the roles of plant hormones, their homeostasis, and associated signaling pathways that contribute to seed formation in *P. neoveitchii* are completely inadequate.

Sugars, such as sucrose and glucose, are required for seed growth. They supply the energy required for a variety of metabolic processes, such as cell division, elongation, and expansion throughout embryonic development (Weber et al., 1997). In recent years, several studies have suggested that RNA sequencing (RNA-seq) is an extremely efficient and robust tool for acquiring substantial transcriptome information in a wide range of plants (Ma et al., 2013; Qi et al., 2014; Li et al., 2016). In comparison to other hardwood species (Tuskan et al., 2006; He et al., 2013; Dai et al., 2014; Liu et al., 2014), conifers have bigger and more repetitive genomes (Birol et al., 2013; Nystedt et al., 2013; Zimin et al., 2014). Even with high-throughput Illumina sequencing technologies, sequencing the *P. neoveitchii* genome remains costly. On the other hand, better transcriptome sequencing data that can reveal information about gene expression regulation during seed formation is valuable. As omics technology is growing rapidly, widely metabolomics analysis has also been widely used to study the regulatory mechanisms of the accumulation of nutrients (Li et al., 2021; Lu et al., 2022). Furthermore, the genes and metabolites were identified by combining transcriptomic and metabolomics results (Chu et al., 2022).

In this study, we compared the seeds from two locations, such as Taibai (Shaanxi province), a 150-year-old tree (TB150), and Zhouqu (Gansu province), a 150-year-old tree (ZQ150), to identify the reasons behind empty seed shell formation at both locations. We measured the endogenous hormone contents, and further, to get global insights into the molecular mechanisms, we performed RNA-seq and widely metabolomics. This study mainly focused on the classification of assembled unigenes involved in plant hormone signaling pathways and sugar metabolism.

## 2 Materials and methods

### 2.1 Sample collection

In this study, the corns of *Picea neoveitchii* Mast. were collected from two provinces of China: Gansu, Zhouqu (ZQ) (E104°24′3″, N33°34′2″) and Shaanxi, Taibai (TB) (E107°30′54″, N33°48′33″). The selected trees were approximately 150 years old at both sites (ZQ150 and TB150), with three trees sampled at each site. Tree ages were estimated with the assistance of local Forestry Bureau staff, who provided informed approximations based on visual inspection and their expertise. With the permission of the Forestry Administration of both sites, a total of 15–20 cones of similar size were harvested in three biological replications on 5 July 2023, at TB and 9 July 2023, at ZQ from the middle of each tree crown to eliminate the possibility of an effect of cone position on the analyzed seed attributes. The harvested samples were maintained following the institutional guidelines of the College of Landscape Architecture and Art, Northwest A&F University, Yangling 712100, China. The same-sized scales at the center of the cones had been removed, and seeds were collected. The collected seeds were then immediately immersed in liquid nitrogen to preserve their integrity before being stored at −80°C for further analysis.

### 2.2 RNA extraction, library preparation, and sequencing

Total RNA was extracted from the seeds of ZQ150 and TB150 by TRIzol^®^ Reagent. Subsequently, RNA quality was determined using a 5300 Bioanalyzer (Agilent) and quantified by the ND-2000 (NanoDrop Technologies). To construct the sequencing library, only high-quality RNA samples (OD260/280 = 1.8–2.2, OD260/230 ≥ 2.0, RIN ≥ 6.5, 28S:18S ≥ 1.0, > 1 μg) were used. RNA purification, reverse transcription, library construction, and sequencing were executed at Shanghai Majorbio Bio-Pharm Biotechnology Co., Ltd. (Shanghai, China) according to the manufacturer’s protocol (Illumina, San Diego, CA). Using 1 μg of total RNA, RNA-seq transcriptome libraries were prepared following Illumina^®^ Stranded mRNA Prep, Ligation from Illumina (San Diego, CA). mRNA was isolated by the polyA selection method using oligo (dT) beads and subsequently fragmented by fragmentation buffer. Then, double-stranded cDNA was synthesized by a SuperScript double-stranded cDNA synthesis kit (Invitrogen, CA) with random hexamer primers (Illumina). After that, the cDNA was subjected to end-repair, phosphorylation, and “A” base addition following Illumina’s library construction method. Libraries were size selected for cDNA target fragments of 300 bp on 2% low-range ultra-agarose, followed by polymerase chain reaction (PCR) amplified by Phusion DNA polymerase (NEB) for 15 PCR cycles. After being quantified using Qubit 4.0, a paired-end RNA-seq sequencing library was sequenced with the NovaSeq 6000 sequencer (2 × 150 bp read length).

### 2.3 Quality control and *de novo* assembly

The raw paired-end reads were trimmed and quality controlled by Fastp (Chen et al., 2018) with default parameters. Then, clean data from the samples were used to do *de-novo* assembly with Trinity (Grabherr et al., 2011). To increase the assembly quality, all the assembled sequences were filtered by CD-Hit and transposed. The assembled transcripts were searched against the National Center for Biotechnology Information (NCBI) protein non-redundant (NR), Clusters of Orthologous Genes (COG), and Kyoto Encyclopedia of Genes and Genomes (KEGG) databases using Diamond to identify the proteins that had the highest sequence similarity with the given transcripts to retrieve their function annotations, and a typical cutoff E-value less than 1.0 × 10−5 was set. The BLAST2GO (Conesa et al., 2005) program was used to get Gene Ontology (GO) annotations of unique assembled transcripts for describing biological processes, molecular functions, and cellular components. Metabolic pathway analysis was performed using the KEGG (Kanehisa and Goto, 2000).

### 2.4 Differential expression analysis and functional enrichment

To identify differentially expressed genes (DEGs) between both samples, the expression level of each transcript was calculated according to the transcripts per million reads (TPMs) method. RSEM (Li and Dewey, 2011) was used to quantify gene abundances. Essentially, differential expression analysis was performed using DESeq2 (Love et al., 2014). DEGs with |log*_2_* FC|≧1 and FDR ≤ 0.05 (DESeq2) were measured to have significantly different expressed unigenes. Furthermore, functional enrichment analyses such as GO and KEGG were executed to find out which DEGs were considerably enriched in GO terms and metabolic pathways at a Bonferroni-corrected *p*-value ≤ 0.05. GO functional enrichment and KEGG pathway analysis were carried out by Goatools and KOBAS (Xie et al., 2011), respectively.

### 2.5 Gene expression analysis

The RT-qPCR assay was conducted on an iCycler iQ Real-Time PCR Detection System (Bio-Rad). The PCR conditions were 95°C for 30 s, followed by 45 cycles of 95°C for 3 s for denaturation and 60°C for 30 s for annealing and extension. The EF1 gene was used as a reference gene (Gong et al., 2021). Relative expression levels of the selected genes were normalized and subsequently calculated by the comparative Ct (2^–ΔΔC^) method (Schmittgen and Livak, 2008). Three biological replications were made, and the sequences of primers are shown in **Supplementary Table S1**.

### 2.6 Metabolite extraction

The process involved weighing a 0.1 *g* sample, grinding it into homogenate in liquid nitrogen, and then re-suspending it with a pre-cooled 80% methanol solution and a 0.1% formic acid solution. Next, the samples were incubated on ice and centrifuged in a low-temperature refrigerated centrifuge. The centrifugation conditions were set as follows: 14,000*g*, 4°C, 30 min. The supernatant in the centrifuge tube was extracted with a pipet gun and diluted with liquid chromatography-mass spectrometry (LC-MS) water to a final concentration of 53% methanol. Then centrifuged again, the centrifugal conditions were the same as above (Want et al., 2013).

### 2.7 HPLC-MS/MS analysis

LC-MS/MS analysis was conducted using an ExionLCTM AD system (SCIEX) coupled with a QTRAP^®^ 6500 + mass spectrometer (SCIEX) in Genedenovo (Guangzhou, China). Injecting the samples into Xselect HSS T3 (2.1 mm × 150 mm, 2.5 μm), using a 20-min linear gradient with a flow rate of 0.4 mL/min for positive/negative polarity mode. The eluent consisted of eluent A (0.1% formic acid water) and eluent B (0.1% formic acid acetonitrile) (Pennington, 2002). The solvent gradient was set as follows: 2% B, 2 min; 2%–100% B, 15.0 min; 100% B, 17.0 min; 100%–2% B, 17.1 min; 2% B, 20 min.

### 2.8 Metabolite identification and quantification

The use of Multiple Reaction Monitoring for the detection of experimental samples was based on a housing database. Using SCIEX OS version 1.4 to process data files generated by HPLC-MS/MS for integration and peak correction. The main parameter settings were as follows: minimum peak height, 500; signal-to-noise ratio, 5; gaussian smoothing width, 1. The area of each peak indicated the relative content of the corresponding substance.

### 2.9 Endogenous hormone extraction

The extraction and purification of endogenous hormones such as IAA, ABA, zeatin riboside (ZR), BR, MeJA, and GA_3_ were also carried out at the Center of Plant Growth Regulator, China Agricultural University. Three biological replications from the seed of TB150 and ZQ150 were assessed. A detailed description of hormone extraction can be found in an earlier paper (Wang et al., 2020).

### 2.10 Statistical analysis

The data were coded, and analysis of variance was done using Statistics 8.1. The treatment means were compared using the least significant difference (LSD) test at *P* ≤ 0.05. The figures were made by GraphPad Prism version 10.00 for Windows GraphPad software (San Diego, California, USA).

## 3 Results

### 3.1 Phenotype of cone and seeds

**Figure 1:**
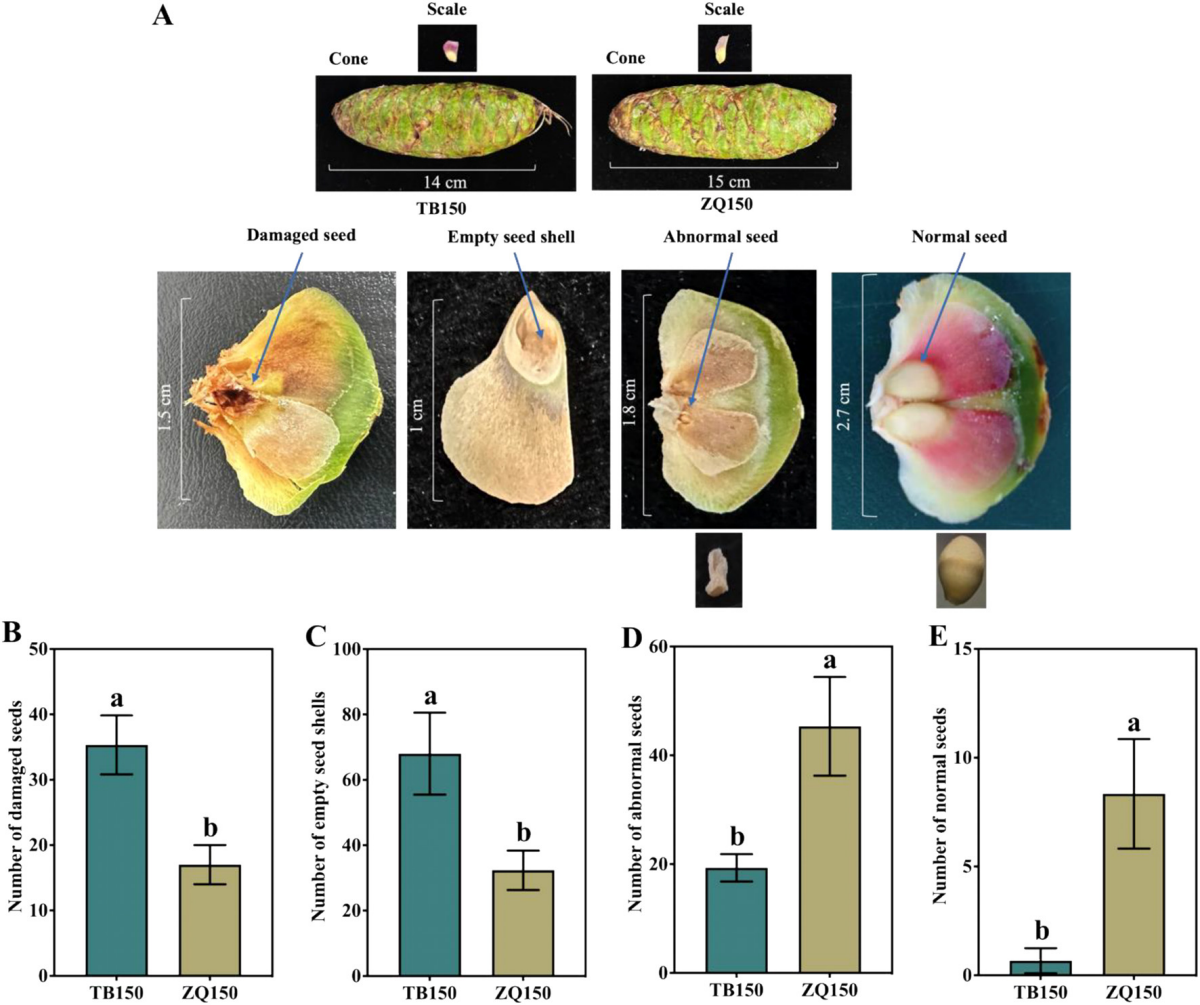
The phenotypes of cones, scales, damaged seeds, empty seed shells, abnormal seeds, and normal seeds in *Picea neoveitchii* Mast. Trees. The scale line represents the actual length in centimeters. **(A)** The number of damaged seeds **(B)**, number of empty seed shells **(C)**, number of abnormal seeds **(D)**, and number of normal seeds **(E)** at Taibai 150-year-old tree (TB150) and Zhouqu 150-year-old tree (ZQ150). Error bars refer to the average value ± SD from three biological replicates. Different letters above the columns indicate significant differences according to a least significant difference (LSD) test at a 0.05% level.

The phenotypes of cones, scales, damaged seeds, empty seed shells, abnormal seeds, and normal seeds are shown in **Figure 1A**. Results suggested that TB150 had the highest percentage of damaged seeds, which was 107.84% higher than ZQ150. Similarly, the percentage of empty seed shells in TB150 was 110.30% higher than the percentage of ZQ150 (**Figures 1B, C**). Moreover, the number of abnormal seeds was higher in ZQ150 as compared to TB150; interestingly, the number of normal seeds was also higher in ZQ150 (**Figures 1D, E**).

### 3.2 RNA-sequencing data quality control

A transcriptomic analysis was conducted to gain deeper insights into the molecular mechanisms behind these inconsistencies. **Supplementary Table S1** represents the statistical summary of three biological repeats. A total of 43–48 million raw and 42–47 million clean reads were obtained. The Q20 and Q30 percentages were more than 95.1 and 92.02, respectively. The percentage of GC content was more than 45.76% (**Supplementary Table S2**). For *de-novo* transcriptomic assembly, **Supplementary Tables S3, S4** present the evaluation of the initial and optimal assembly results. The Trinity program assembled a total of 66,445 unigenes based on high-quality reads (**Supplementary Table S4**). The highest number of unigenes were found in the 200–500 sequence length distribution, and 501–1000 was at the second (**Supplementary Figure S1**). Furthermore, 21.19–23.77 million clean reads, 18.66–20.72 million mapped reads, and an 87.15%–88.71% mapped ratio was observed in a comparison of sequencing data and assembly results (**Supplementary Table S5**).

### 3.3 Transcriptome functional annotation

To perform the annotation of the generated unigenes, alignment searches were performed against publicly available bioinformatics databases. The results suggested that 33,568 (50.51%) unigenes out of 66,445 unigenes had strong similarity to proteins in the NCBI NR database. Similarly, 40.62%, 17.37%, 38.39%, 34.53%, and 34.99% unigenes had similarities in the GO, KEGG, clusters of orthologous groups (eggNOG), swiss-prot protein database (Swiss-Prot), and protein families database (Pfam), respectively (**Table 1**).

**Table 1:** Summary of unigene annotation.

Annotation database	Number of annotated unigenes	Percentage of annotated unigenes
GO	26994	40.62%
KEGG	11545	17.37%
eggNOG	25514	38.39%
NR	33568	50.51%
Swiss-Prot	22945	34.53%
Pfam	23250	34.99%

NR, NCBI Non-Redundant Protein Database; Swiss-Prot, Swiss-Prot Protein Database; GO, Gene Ontology; KEGG, Kyoto Encyclopedia of Genes and Genomes Pathway.

To further assess the functional distribution of our data, GO and KEGG annotation analyses were used. Based on sequence homology, 26994 (40.62%) unigenes were classified into GO terms. Cellular process (48.63%) and catalytic activity (44.97%) were dominant; we also noticed that the metabolic process (42.89%), cell part (41.38%), and membrane part (30.63%) had a representation of unigenes (**Supplementary Figure S2A**). The KEGG database encompasses a comprehensive collection of pathways and networks that govern molecular regulation within cells. It establishes connections between genes and gene products within these pathways. Depending on the comparison, 11,545 (17.37%) unigenes were annotated against the KEGG database. Carbohydrate metabolism, lipid metabolism, and amino acid metabolism contain the highest number of unigenes in metabolism. In genetic information processing; folding, sorting and degradation, translation, and transcription were the most enriched pathways. Moreover, environmental adaptation, signal transduction, and transport and catabolism were heavily enriched in organismal systems, environmental information processing, and cellular processes, respectively (**Supplementary Figure S2B**).

### 3.4 Identification of DEGs and functional enrichment analysis

**Figure 2:**
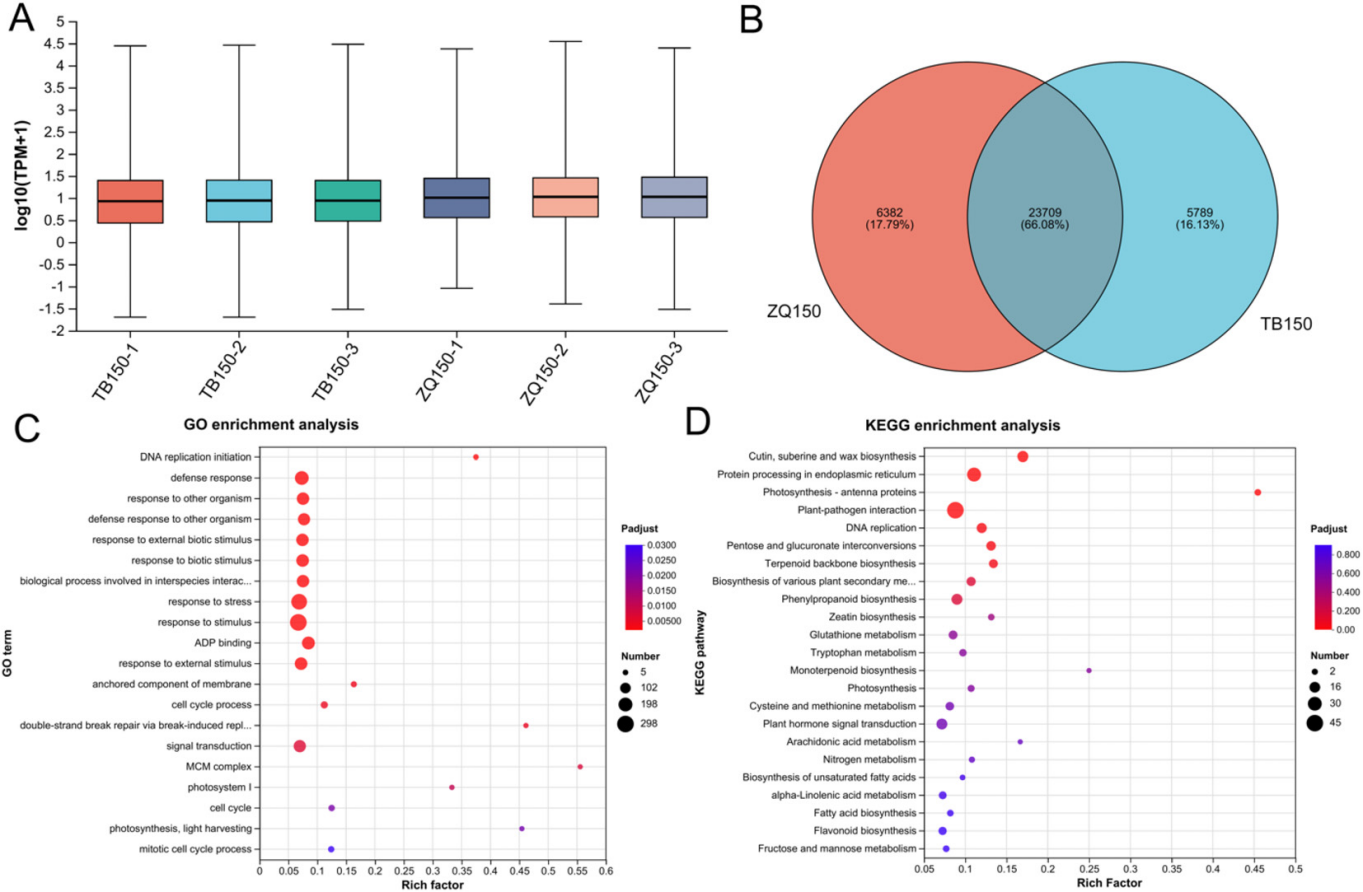
The boxplot showed the unigene expression distribution of each sample **(A)**. Venn analysis represents the number of shared and unique unigenes among both samples **(B)**. Gene classification was based on Gene Ontology (GO) **(C)** and Kyoto Encyclopedia of Genes and Genomes (KEGG) analysis for DEGs **(D)**. The GO term and KEGG pathway names are provided on the vertical axis. The horizontal axis represents the Rich factor (the ratio of the number of unigenes enriched in the GO term and KEGG pathways to the number of annotated genes). The larger the rich factor, the greater the degree of enrichment. The size of the dot represents the number of genes, and the color of the dot corresponds to different false discovery rate (FDR) (Pvaule_corrected) ranges.

The expression distribution of unigenes in each sample is shown in **Figure 2A**. The Venn analysis showed the shared and uniquely expressed unigenes in TB150 and ZQ150, where 6,382 unigenes were uniquely expressed in ZQ150 and 5,789 unigenes were uniquely expressed in TB150, with a shared 23,709 unigene in both groups (**Figure 2B**). Furthermore, in order to simplify the data and examine the relationship and variance between samples more thoroughly, we employed principal components analysis (PCA). We observed an obvious divergence between the two samples (**Supplementary Figure S2C**). A total of 2,355 DEGs were found between TB150 and ZQ150, with 1,280 upregulated and 1,075 downregulated unigenes. The volcano specifies the DEGs (**Supplementary Figure S2D**; **Additional File 2**).

Furthermore, we also used GO and KEGG functional enrichment analyses to figure out the underlying biological processes and key functions that are significant in DEGs. A total of 24 GO terms (18 biological process, 4 cellular component, and 2 molecular function) were perceived between both samples (**Figure 2C**; **Supplementary Table S6**). The mainly affected biological process category includes response to stimulus (GO:0050896), response to stress (GO:0006950), and defense response (GO:0006952). Furthermore, the anchored component of membrane (GO:0031225) and photosystem I (GO:0009522) were primarily affected in the cellular component category. Moreover, adenosine diphosphate binding (GO:0043531) and oxidoreductase activity, acting on CH or CH2 groups (GO:0016725) were only affected in the molecular function category (**Figure 2C**; **Supplementary Table S6**). The KEGG pathway enrichment analysis indicated that plant–pathogen interaction (map04626), protein processing in the endoplasmic reticulum (map04141), plant hormone signal transduction (map04075), DNA replication (map03030), pentose and glucuronate interconversions (map00040), and starch and sucrose metabolism (map00500) were the most enriched pathways (**Figure 2D**; **Supplementary Table S7**).

### 3.5 Identification of differential metabolites and functional enrichment analysis

**Figure 3:**
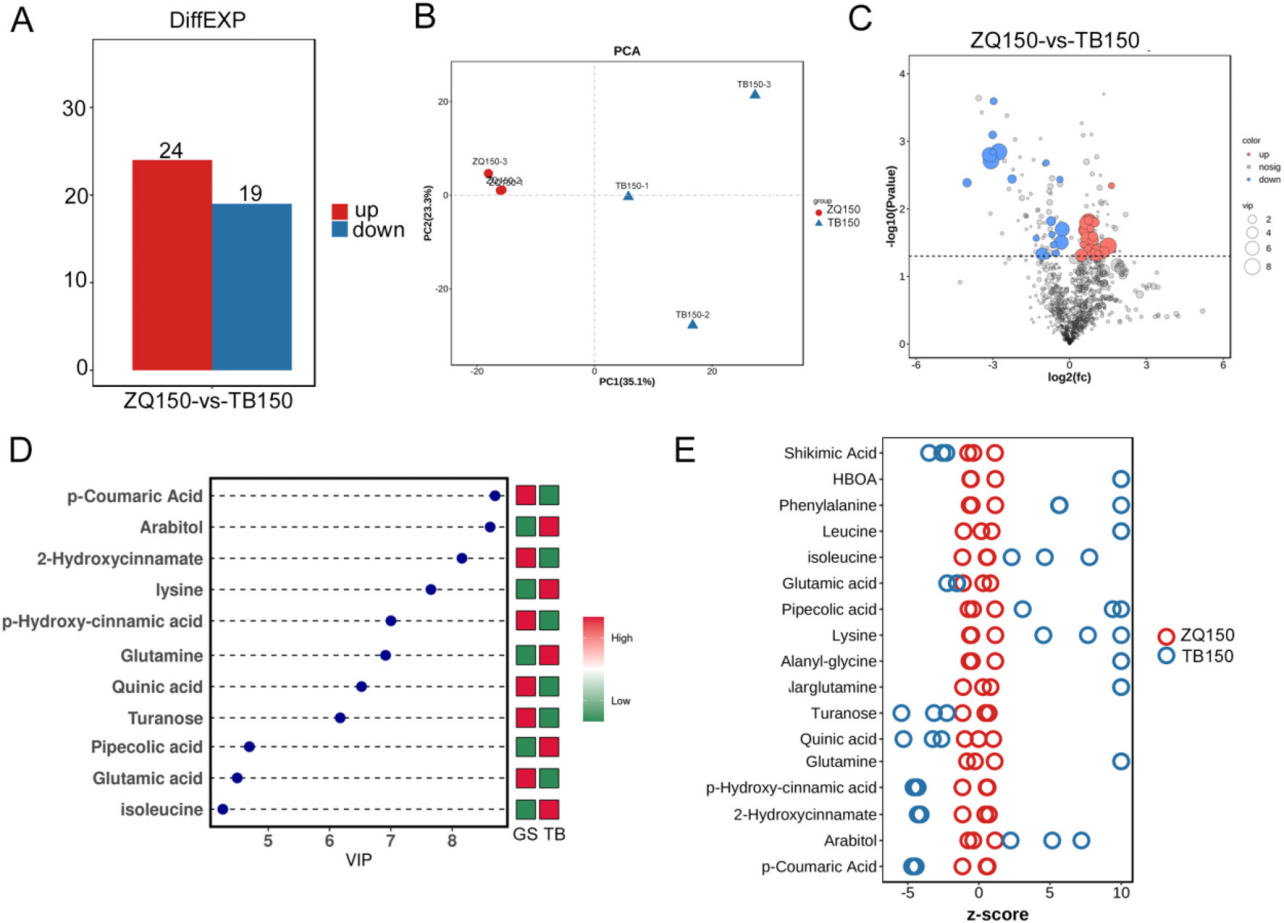
Metabolomics analysis. Statistical chart of differential metabolite quantity **(A)**. Quality control principal components analysis (PCA) diagram **(B)**. Volcano plots of differential metabolites among TB150 versus ZQ150 **(C)**. Variable Importance in Projection (VIP) of orthogonal partial least squares discriminant analysis (TB150 versus ZQ150) **(D)**. Z-score plot of differential metabolites **(E)**.

We applied the LC-MS–based metabolomics method to conduct extensive compound analysis of TB150 and ZQ150 to study their metabolite differences. TB150 and ZQ150 showed obvious changes at 43 different metabolites (**Figures 3A, C**). PCA, orthogonal partial least squares discriminant analysis, and partial least squares discriminant analysis all show that the sample distribution was normal, with notable differences (**Figure 3B**; **Supplementary Figures S3A–C**). Heat maps of metabolite differences between them clearly showed a significant difference (**Supplementary Figure S3D**), and their correlation was also reasonable (**Supplementary Figure S3E**). It was also found that there were more differences in metabolic groups between TB150 and ZQ150. Among them, arabitol, glutamine, and lysine were significantly upregulated (**Figure 3D**). The Z-score plot of differential metabolites identified a significantly different compound as p-coumaric acid, various amino acids (glutamine, p-hydroxy-cinnamic acid, 2-hydroxycinnamate), and sugars (turanose) (**Figure 3E**).

In addition, the KEGG pathway was assigned to study the biological processes associated with differential metabolites. A KEGG analysis was performed on all the identified compounds, and the results showed that the metabolites mainly include hormone metabolism and secondary metabolism (**Supplementary Figure S4A**). The differential metabolites of TB150 and ZQ150 mainly took part in the phenylalanine metabolism, 2-Oxocarboxylic acid metabolism, tyrosine metabolism, 2-oxocarboxylic acid metabolism, and so on (**Supplementary Figure S4B**). Metabolite Set Enrichment Analysis (MSEA) identified and explained the patterns of changes in metabolite concentrations in some important biological pathways. MSEA of TB150 and ZQ150 causes significant changes in phospholipid biosynthesis, lysine degradation, and amino sugar metabolism (**Supplementary Figure S4C**).

### 3.6 The metabolome and transcriptome are coregulated between TB150 and ZQ150

**Figure 4:**
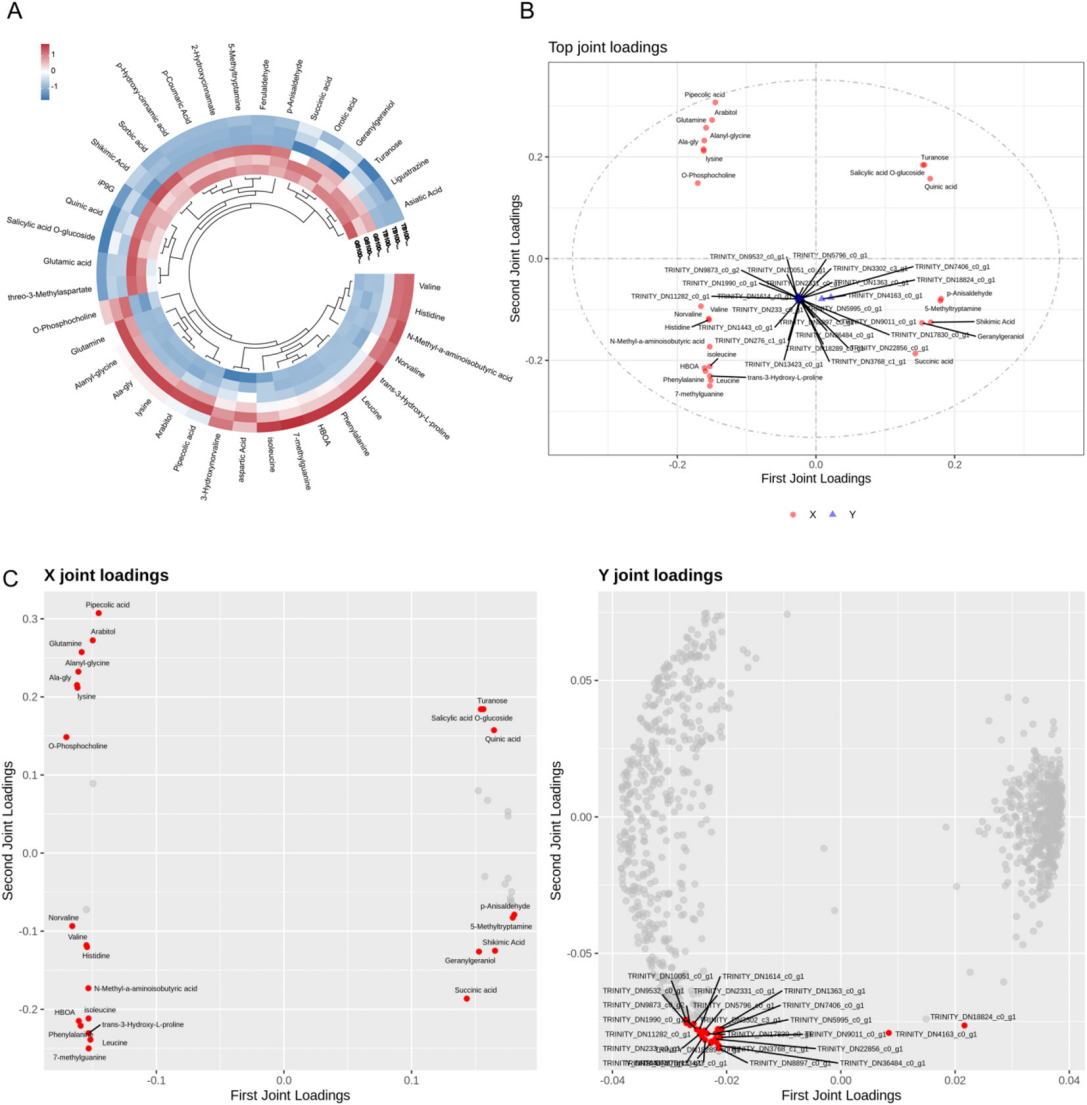
Metabolic pathway analysis. Kyoto Encyclopedia of Genes and Genomes (KEGG) pathway assignment of all metabolites among TB150 versus ZQ150 **(A)**. KEGG pathway assignment of differential metabolites among TB150 versus ZQ150 **(B)**. MSEA enrichment map **(C)**.

The differential metabolite heatmap analysis reveals 43 metabolites in TB150 and ZQ150 (**Figure 4A**). We used the transcriptome and metabolomics of each dataset for an integrated analysis. More than 90% of the total variation in the metabolic (R^2^X = 0.979) and metabolic transcriptome groups (R^2^Y = 0.961) was explained using the model of three combined components. After calculating the load coefficient threshold after 1,000 permutations, we determined that 929 transcripts and 43 metabolites had the greatest impact on the model (**Figure 4B**). The main transcripts included TRINITY_DN36484_c0_g1, TRINITY_DN8897_c0_g1, TRINITY_DN3768_c1_g1, TRINITY_DN18289_c0_g1, TRINITY_DN22856_c0_g1, TRINITY_DN13423_c0_g1, and so on. Pipecolic acid, D-phenylalanine, D-arabitol, 7-methylguanine, glutamine, and D-glutamine were also some of the main metabolites (**Figure 4C**).

### 3.7 Endogenous hormone contents and expression of hormone signaling pathway*–*related genes

The content of endogenous hormones such as IAA, ABA, BR, MeJA, ZR, and GA_3_ was measured in both groups. The number of DEGs involved in the plant hormone signal transduction pathway was also identified. A total of 18 DEGs were annotated in the plant hormone signal transduction pathways: auxin, ABA, BR, JA, salicylic acid (SA), and ethylene. The details are given below.

#### 3.7.1 Measurement of IAA and ABA contents and expression analysis of related DEGs

**Figure 5:**
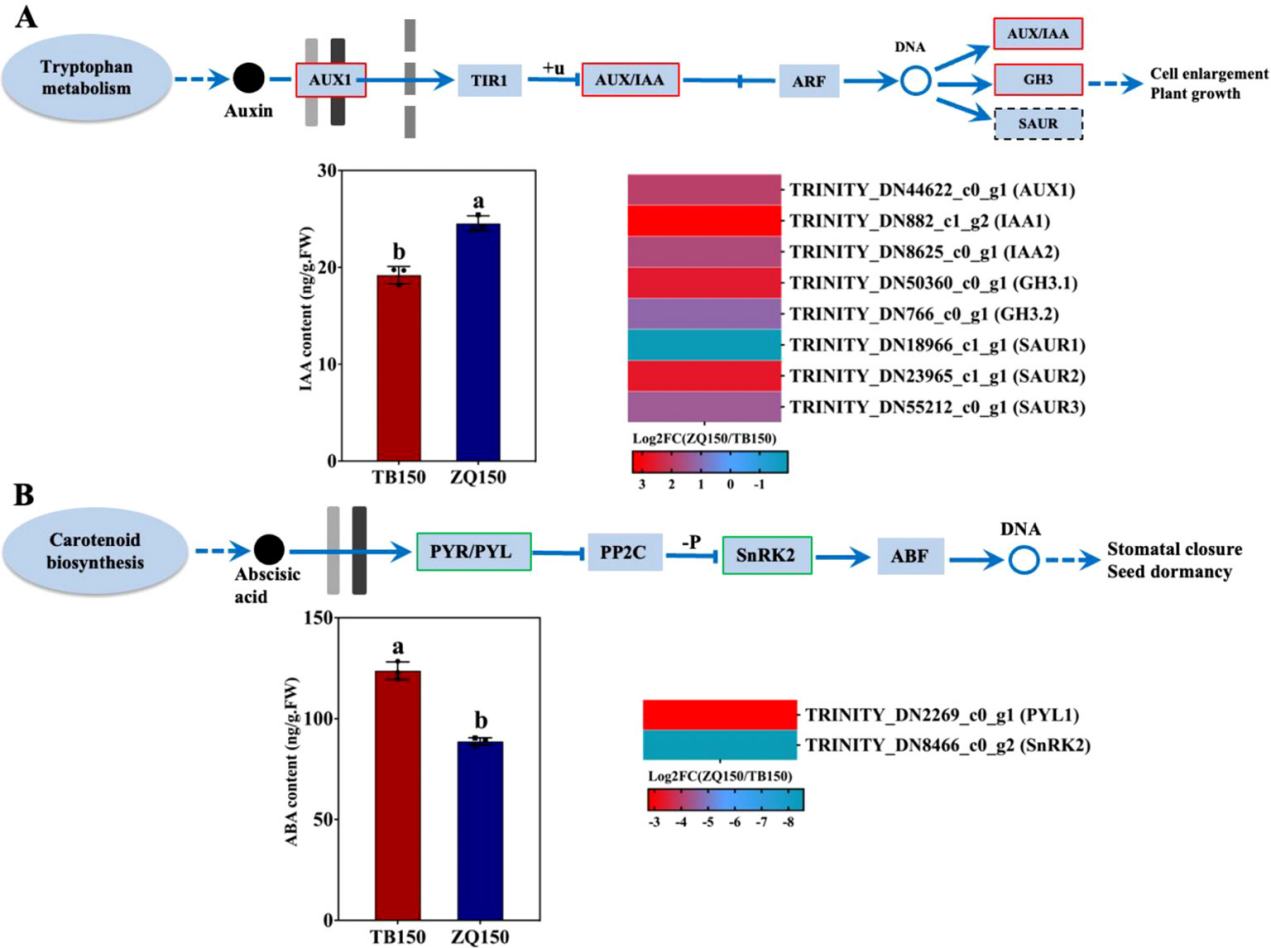
Measurement of the endogenous content of indole-3-acetic acid (IAA) and selected differentially expressed genes (DEGs) related to auxin from the RNA sequencing data **(A)**. The concentration of abscisic acid (ABA) and selected DEGs related to ABA **(B)**. Heat map diagram of the log_2_FC; the color scale represents the expression levels from lower to higher. Error bars refer to the average value ± SD from three biological replications. Different letters above the columns indicate significant differences according to a least significant difference (LSD) test at a 0.05% level.

The endogenous content of IAA was found to be higher in ZQ150 as compared to TB150. Furthermore, one auxin influx carrier, *AUX1*, was upregulated. The AUX/IAA transcriptional regulator family proteins, *IAA1* and *IAA2*, were also upregulated. Two members of the auxin-responsive GH3 family protein, such as *GH3.1* and *GH3.2*, were also upregulated. Three members of the small auxin upregulated RNA (SAUR) SAUR-like auxin-responsive protein family were identified, of which *SAUR1* was downregulated and the other two, *SAUR2* and *SAUR3*, were upregulated (**Figure 5A**). In addition, the ABA concentration was opposite in trend with IAA, where ABA was elevated in TB150 as compared to ZQ150. Only two DEGs related to the ABA receptor *PYL1* and the ABA response factor *SnRK2* were found; the expression of both was downregulated (**Figure 5B**).

#### 3.7.2 Measurement of BR and JA contents and expression analysis of related DEGs

**Figure 6:**
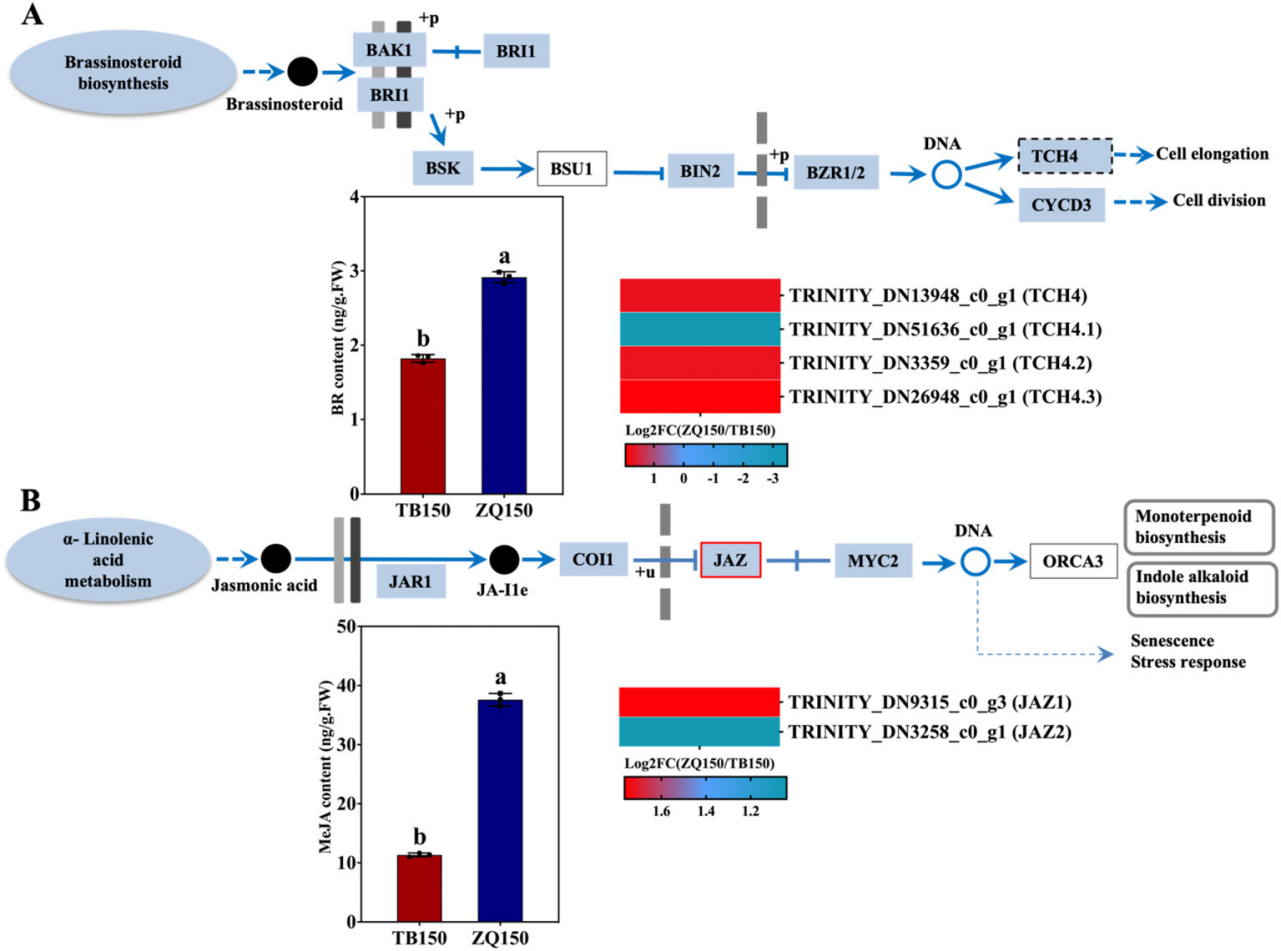
Measurement of the endogenous content of brassinosteroid (BR) and selected differentially expressed genes (DEGs) related to BR from the RNA-sequencing data **(A)**. The concentration of methyl jasmonate (MeJA) and selected DEGs related to JA **(B)**. Heat map diagram of the log_2_FC; the color scale represents the expression levels from lower to higher. Error bars refer to the average value ± SD from three biological replications. Different letters above the columns indicate significant differences according to a least significant difference (LSD) test at a 0.05% level.

The endogenous concentration of BR was observed to be greater in the ZQ150 in comparison to the TB150. The expression of BR-related genes annotated with BR signaling pathways was also investigated. The expressions of the Xyloglucan endotransglucosylase/hydrolase family proteins *TCH4, TCH4.1, TCH4.2*, and *TCH4.3* were found to be upregulated in ZQ150 except TCH4.1 (**Figure 6A**). Furthermore, the concentration of JA was also higher in ZQ150 as compared to TB150. Only two DEGs were found in the JA signaling pathway, jasmonate-zim-domain protein (*JAZ1* and *JAZ2*), and both were also upregulated in ZQ150 (**Figure 6B**).

#### 3.7.3 Measurement of ZR and GA_3_ contents and expression analysis of related DEGs

**Figure 7:**
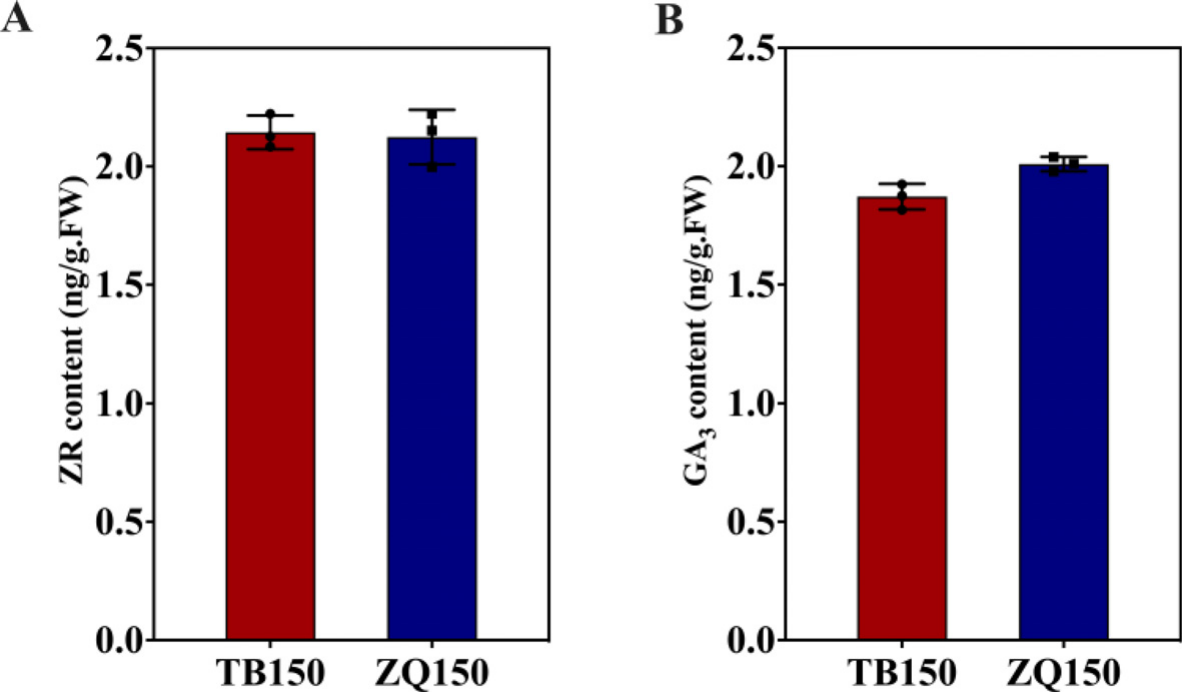
Measurement of the endogenous contents of zeatin riboside (ZR) **(A)** and gibberellic acid 3 (GA_3_) ***(*B*)***. Error bars refer to the average value ± SD from three biological replications.

In this study, the concentrations of ZR and GA_3_ were also measured in both groups. We did not notice any difference in the content of ZR between both groups (**Figure 7A**). However, GA_3_ contents were slightly higher in ZQ150 as compared to TB150 (**Figure 7B**). Unfortunately, we did not find any DEGs related to ZR and GA_3_ in our analysis.

#### 3.7.4 Expression analysis of SA- and ethylene-related DEGs

**Figure 8:**
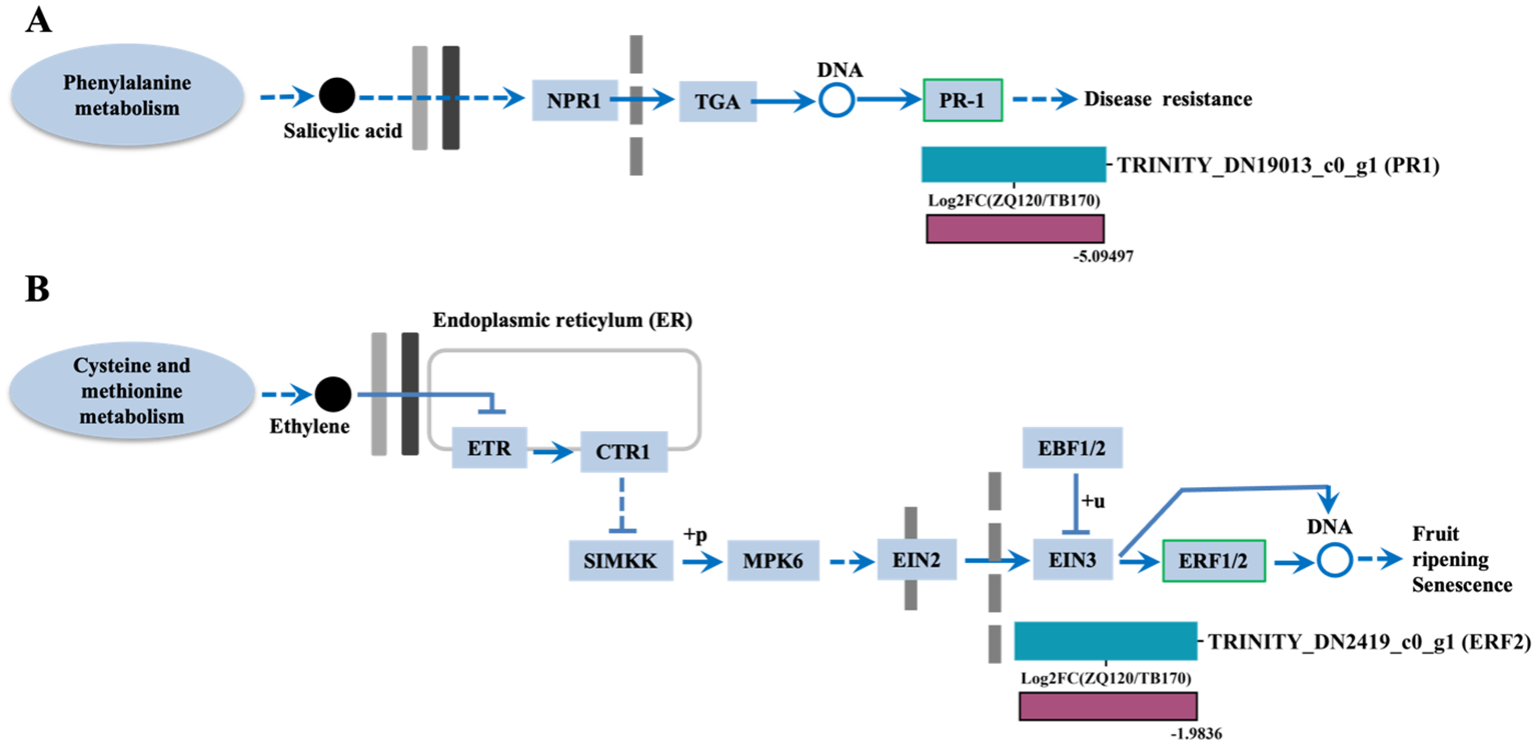
Selected differentially expressed genes (DEGs) related to salicylic acid (SA) **(A)** and ethylene **(B)** were identified from the RNA sequencing data. Heat map diagram of the log*_2_* FC; the color scale represents the expression levels from lower to higher.

We only found two DEGs related to SA and ethylene, such as pathogenesis‐related protein‐1‐like (PR-1 and ethylene response factor 1/2 (ERF1/2). The expressions of both *PR1* and *ERF2* were downregulated in ZQ150 (**Figures 8A, B**).

### 3.8 Expression analysis of sugar metabolism-related DEGs

Sugar content and metabolism were associated with the status of energy supply at any developmental stage. In this study, the expression regulations of starch and sucrose, fructose and mannose, glycolysis/gluconeogenesis, and the TCA cycle were also measured. As shown in the list of sugar metabolism-related genes, the highest numbers of DEGs (12) were found in starch and sucrose. The expression level of most DEGs was downregulated, and only four DEGs were upregulated (TRINITY_DN11824_c0_g2, TRINITY_DN12787_c0_g1, TRINITY_DN4613_c0_g1, and TRINITY_DN905_c0_g3) (**Supplementary Figure S5**). In fructose and mannose, three DEGs (TRINITY_DN10225_c0_g1, TRINITY_DN19840_c0_g1, and TRINITY_DN25234_c0_g2) were downregulated, and two DEGs, including TRINITY_DN905_c0_g3 and TRINITY_DN989_c0_g1, were upregulated (**Supplementary Figure S5**). All DEGs in glycolysis were downregulated except two, such as TRINITY_DN39231_c0_g2 and TRINITY_DN54133_c0_g1 (**Supplementary Figure S5**). TRINITY_DN783_c0_g1 was only found in TCA, and it was downregulated (**Supplementary Figure S5**).

### 3.9 Validation of RNA-seq data by RT-qPCR

**Figure 9:**
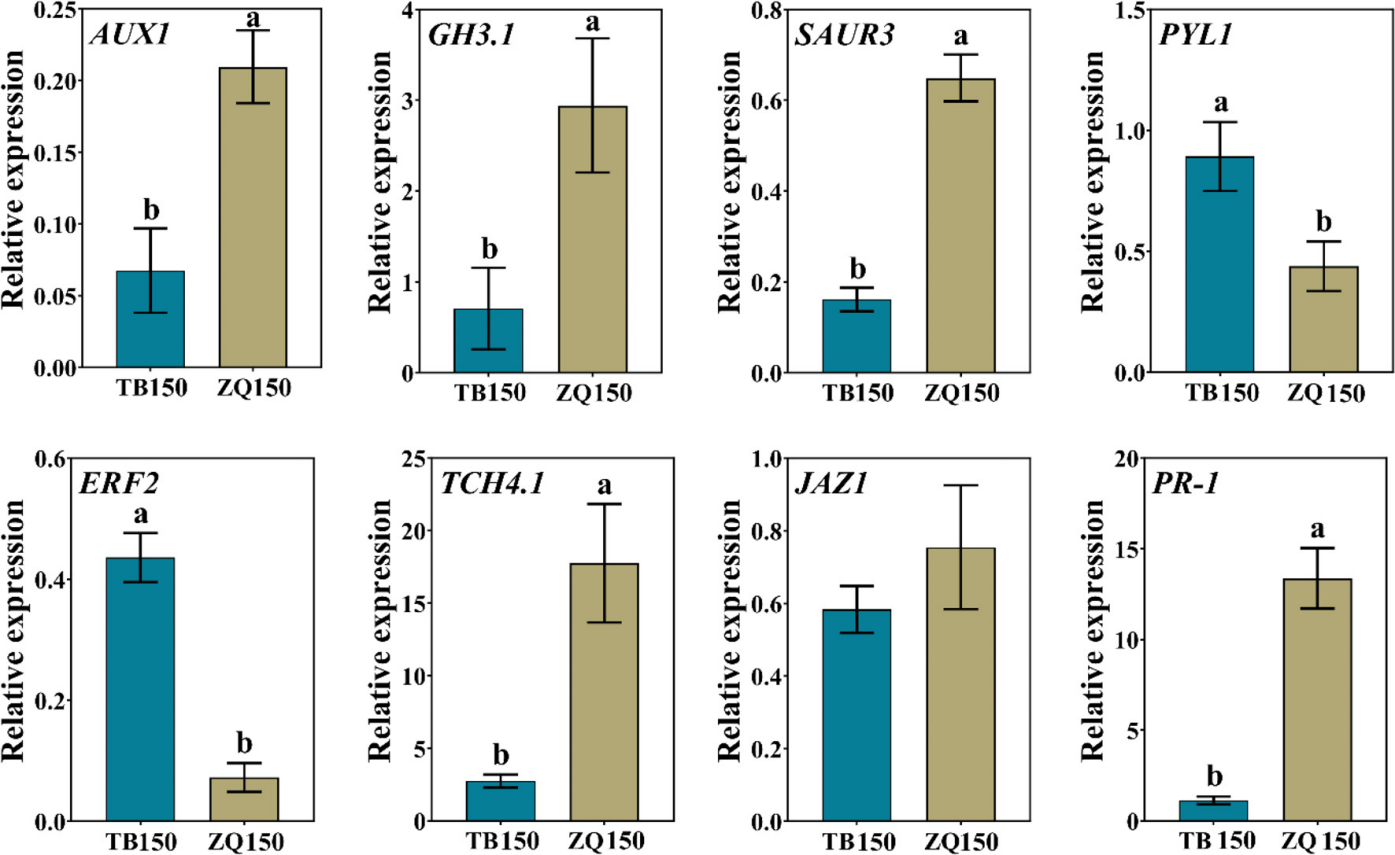
RT-qPCR–based expression analysis of differentially expressed genes (DEGs) to authenticate the RNA-seq data. Error bars refer to the average value ± SD from three biological replications. Different letters above the columns indicate significant differences according to a least significant difference (LSD) test at a 0.05% level.

In order to validate our data, eight DEGs related to different hormones were selected for expression analysis using RT-qPCR. The results suggested that the RT-qPCR–based gene expression patterns of all DEGs were closely parallel to those perceived from RNA-seq data, except *PR-1*, whose expression was not consistent with the RNA-seq data (**Figure 9**). Therefore, we believe that the RNA-seq data are reliable.

## 4 Discussion

### 4.1 Empty seed shell formation in different locations is significantly affected by endogenous hormones and sugar

*P. neoveitchii* propagation chiefly depends on seeds, and the process of seed formation and development is highly complex and takes several months, affecting various factors, including endogenous hormones, sugar, and related gene expression. However, empty seed shells are a common problem that *P. neoveitchii* trees face in China, and simultaneously, the intensity of empty seed shells varies at different locations. In this study, we found that TB150 had the highest percentage of damaged seeds and empty seed shells than ZQ150, and ZQ150 developed a higher number of normal (viable) seeds as compared to TB150 (**Figure 1**). To determine the physiological and molecular changes behind these inconsistencies, we determined the transcriptomic and metabolomic changes during site-dependent seed formation in *P. neoveitchii* trees.

### 4.2 Molecular insights into the site-dependent changes in seed formation and metabolism

The study’s findings provide insight into the intricate molecular mechanisms underlying the site-specific variations during seed formation. The results of this study suggested the dynamic interplay between transcriptome and metabolomic responses to tree site-dependent changes by revealing major variations in metabolite profiles and gene expression between TB150 and ZQ150 *P. neoveitchii* trees. Many unigenes showed similarities to known proteins, highlighting the genetic diversity of this species. Transcriptome functional annotation revealed a wealth of genomic resources. The 2355 DEGs found between TB150 and ZQ150 suggest a significant degree of transcriptional alteration associated with the location. Furthermore, enrichment analysis revealed pathways that were important for site-specific seed development, including plant-pathogen interaction, hormone signaling (e.g., plant hormone signal transduction), as well as metabolic activities (e.g., glucose metabolism).

Moreover, LC-MS–based metabolomics identified 43 metabolites with variable abundance levels between TB150 and ZQ150 (**Figures 3A, C**). Among these were markedly elevated levels of metabolites, including arabitol, glutamine, and lysine (**Figure 3D**). The pathway enrichment analysis (**Figure 4A**) revealed changes in the metabolism of amino acids, carbohydrates, and secondary metabolism, indicating site-specific changes in metabolism during seed growth (Baud and Lepiniec, 2009). In addition, the integration of transcriptomic and metabolomic datasets revealed the coregulated networks that were found between genes and metabolites, showing the specific transcripts, including TRINITY_DN36484_c0_g1, TRINITY_DN8897_c0_g1, TRINITY_DN3768_c1_g1, TRINITY_DN18289_c0_g1, TRINITY_DN22856_c0_g1, TRINITY_DN13423_c0_g1, and so on, and metabolites, including pipecolic acid, phenylalanine, arabitol, 7-methylguanine, and D-glutamine, as vital components in site-related seed metabolism (**Figure 4C**) (Zhang et al., 2017).

In the widely target metabolome, the KEGG analysis was performed on all identified compounds, and the results showed that the metabolites mainly include hormone metabolism and secondary metabolism. Endogenous hormones IAA, ABA, GA, and ZR had certain effects on the seed germination of *Cornus officinalis* (Sheikhzadeh et al., 2021). Differential metabolites identified a significantly different compound as sugar (turanose). Sugar starvation promotes the germination of *Lupinus* spp. seeds by destroying lipid decomposition (Borek et al., 2023). Endogenous hormones and sugar significantly affect the formation of empty seed shells.

### 4.3 Endogenous hormones and related DEGs contribute to empty seed shells

Phytohormones are synthesized inside the plant and act as signaling agents, controlling a variety of events. Physiological activities are triggered by plant hormone signals, which are recognized and carried to the nucleus via signal transduction (Yao et al., 2018). The DEGs were engaged in all of the phytohormone signal transduction pathways; however, we primarily focus on the auxin signaling pathway, which might be responsible for the higher normal seed growth in ZQ150. Auxin is a mobile signaling molecule that controls numerous processes in plant development. Megagametogenesis has been demonstrated to be significantly regulated by auxin. *YUCCA1* (*YUC1*) and *AUX1* genes, which are associated with auxin production and auxin influx carriers, suggest a complex auxin mechanism that is involved in mitotic divisions, cell growth, and patterning during embryonic sac development (Panoli et al., 2015). These results were in agreement with our results, where we found that higher IAA production and induced *AUX1* expression were found in ZQ150, which might be responsible for the high rate of normal seed formation (**Figures 1**, **5A**). Auxin primary response genes, such as *GH3*, *SAUR*, and *AUX/IAA*, were differently expressed at various developmental periods (Yao et al., 2018). In our data, two genes of each *AUX/IAA* and *GH3* were expressed, and their expressions were upregulated in ZQ150; at the same time, three *SUAR* genes were also expressed, where the expression was also induced in ZQ150 except one gene (**Figure 5A**). It is hypothesized that the lower IAA content and reduced expression of auxin signaling pathway genes contributed to empty seed shell growth in TB150 as compared to ZQ150.

Auxin can promote cell division and growth, while ABA is a plant growth inhibitor (Bao and Zheng, 2005). It is thought that ABA-mediated signaling plays a role in plant development, seed germination, and the response to stress. *PYL*, the ABA receptor in the ABA signal transduction pathway, inhibits *PP2C* and is the source of ABA negative regulation (Park et al., 2009). However, an excessive concentration or distribution of ABA in its signaling pathways can result in abnormalities in seed development, such as seed abortion or the production of non-viable seeds (Cutler et al., 2010). In our study, the endogenous content of ABA was found to be higher in TB150 as compared to ZQ150, and the expression of *PYL1* and *SnRK2* was also induced in TB150 (**Figure 5B**). These results suggest that excessive ABA contents may be related to the formation of empty seed shells; however, more research is needed to elucidate the role of hormones in empty seed shell formation in *P. neoveitchii* trees.

### 4.4 Sugar metabolism-related genes contributed to seed formation

In addition to being a crucial route during reproductive growth, energy metabolism controls important movement throughout the plant. There is evidence linking the development of the reproductive system to many regulatory genes involved in the metabolism of starch, sucrose, and glycolysis. *SUS* controls bud primordia count and promotes bud development (Ito et al., 2002). It was found in a previous study that the *SUS* and *AGPL* expression levels affect the seed numbers, and in male fertile rice lines, these expression levels are noticeably lower (Kong et al., 2007). In this study, 12 DEGs were found related to starch and sucrose metabolism, from which the expression of 8 unigenes was induced in ZQ150. Two unigenes out of five were induced in fructose metabolism. In glycolysis, two unigenes were repressed and four unigenes were induced; only one unigene related to TCA was also induced in ZQ150. These results suggest that sugar metabolism plays a key role in the formation of seeds to provide energy during the process; at the same time, lower energy may cause an abnormal or empty seed shell formation in *P. neoveitchii*. However, more work is required to establish their roles in seed formation.

## 5 Conclusion

In this study, we found that different locations had different seed-forming abilities in *P. neoveitchii*. ZQ150 developed a higher number of normal seeds by increasing endogenous IAA and the expression of auxin signaling pathway genes. At the same time, TB150 had the highest percentage of empty seed shells that were related to the increased ABA contents and induced ABA signaling pathway genes. Furthermore, we also suggest that higher expression of sugar metabolism-related genes contributes to normal seed development. Our study provides the foundation for further studies.

## Data Availability

The datasets presented in this study can be found in online repositories. The names of the repository/repositories and accession number(s) can be found below: https://www.ncbi.nlm.nih.gov/,PRJNA1145144.
