# Supplementary material for: Unraveling site-specific seed formation abnormalities in Picea neoveitchii Mast. trees via widely metabolomic and transcriptomic analysis
Source: Front Plant Sci. 2024 Dec 10;15:1495784. doi: 10.3389/fpls.2024.1495784 (PMC11667104; doi:10.3389/fpls.2024.1495784)
Supplement: Supplementary file 1 [file DataSheet1.zip › Data Sheet 1/Data Sheet 1 (13)/Supplementary data/Additional file 1.docx]

**Table S1.** Gene-specific primers used in qRT-PCR analysis

| Gene Name | Sequence |
| --- | --- |
| *GH3.1-F* | ATGCCTCGAGCAAAGAGA |
| *GH3.1-R* | CTAAGTTAACCGAGTCAT |
| *SAUR3-F* | GCGAGAACACATCTTGTGT |
| *SAUR3-R* | TAAATATAATACTCATTTG |
| *AUX1-F* | GGAACTTCCATTATCTTC |
| *AUX1-R* | ACATATGTGTTCTTTAT |
| *PYL1-F* | CGACCATAAGAGTAGTC |
| *PYL1-R* | TGAGATATCAATATTAT |
| *ERF2-F* | GAGGAAACGGAAATCAAT |
| *ERF2-R* | TCAATTATAGCAATCTCA |
| *TCH4.1-F* | ATGGCCAAATATTTCATC |
| *TCH4.1-R* | TTATGTGCTCCTGCATTC |
| *JAZ1-F* | GATTCAGCTCTTTCATTG |
| *JAZ1-R* | ATAAACAGAGTTGTGAAT |
| *PR1-F* | CCAGAGAGTCAATTGCTT |
| *PR1-R* | CACCTCGAGATCGTTCGC |
| *EF1-F* | GCAAGGAACTGGAGAAGGAACC |
| *EF1-R* | GACGCATGTCCCTCACAGCAA |

**Table S2. Sequencing data statistics**

| Sample | Raw reads | Raw bases | Clean reads | Clean bases | Error rate(%) | Q20 (%) | Q30 (%) | GC content (%) |
| --- | --- | --- | --- | --- | --- | --- | --- | --- |
| TB150_1 | 43051692 | 6.5E+09 | 42398090 | 6.22E+09 | 0.0266 | 96.51 | 94.4 | 45.76 |
| TB150_2 | 44422154 | 6.71E+09 | 43540636 | 6.4E+09 | 0.0299 | 95.1 | 92.02 | 45.92 |
| TB150_3 | 48259250 | 7.29E+09 | 47556140 | 6.98E+09 | 0.0265 | 96.54 | 94.44 | 45.85 |
| ZQ150_1 | 44153582 | 6.67E+09 | 43349148 | 6.37E+09 | 0.0269 | 96.39 | 94.13 | 45.96 |
| ZQ150_2 | 44817776 | 6.77E+09 | 44165910 | 6.5E+09 | 0.0267 | 96.43 | 94.29 | 45.88 |
| ZQ150_3 | 44109996 | 6.66E+09 | 43154294 | 6.35E+09 | 0.0277 | 96.04 | 93.52 | 46.05 |

**Table S3.** Evaluation of initial assembly results

| Type | Unigene | Transcript |
| --- | --- | --- |
| Total number | 75779 | 151271 |
| Total base | 69999514 | 170173931 |
| Largest length (bp) | 15355 | 15355 |
| Smallest length (bp) | 201 | 201 |
| Average length (bp) | 923.73 | 1124.96 |
| N50 length (bp) | 1787 | 2013 |
| E90N50 length (bp) | 2620 | 2368 |
| Fragment mapped percent(%) | 75.595 | 89.135 |
| GC percent (%) | 41.66 | 41.76 |
| TransRate score | 0.30007 | 0.29273 |
| BUSCO score | C:81.3%[S:75%;D:6.3%] | C:86.4%[S:45.4%;D:41.0%] |

**Table S4.** Evaluation of optimal assembly results

| Type | Unigene | Transcript |
| --- | --- | --- |
| Total number | 66445 | 108551 |
| Total base | 67013138 | 1.18E+08 |
| Largest length (bp) | 13072 | 13072 |
| Smallest length (bp) | 201 | 201 |
| Average length (bp) | 1008.55 | 1088.23 |
| N50 length (bp) | 1846 | 1839 |
| E90N50 length (bp) | 2616 | 2406 |
| Fragment mapped percent(%) | 25.031 | 87.088 |
| GC percent (%) | 41.71 | 41.87 |
| TransRate score | 0.06689 | 0.40989 |
| BUSCO score | C:80.5%[S:74.2%;D:6.3%] | C:85.6%[S:56.9%;D:28.7%] |

**Table S5.** Comparison of sequencing data and assembly results

| Sample | Clean reads | Mapped reads | Mapped ratio |
| --- | --- | --- | --- |
| TB150_1 | 21199045 | 18666380 | 88.05% |
| TB150_2 | 21770318 | 19097713 | 87.72% |
| TB150_3 | 23778070 | 20722054 | 87.15% |
| ZQ150_1 | 21674574 | 19080632 | 88.03% |
| ZQ150_2 | 22082955 | 19590281 | 88.71% |
| ZQ150_3 | 21577147 | 18977248 | 87.95% |

**Table S6.** GO enrichment analysis

| Num | GO ID | Term | Description | Ratio_in_study | Ratio_in_pop | Pvalue_uncorrected | Pvalue_corrected |
| --- | --- | --- | --- | --- | --- | --- | --- |
| 9 | GO:0006270 | BP | DNA replication initiation | 9/1349 | 24/26994 | 1.25E-06 | 0.002352961 |
| 194 | GO:0006952 | BP | defense response | 194/1349 | 2648/26994 | 3.89E-06 | 0.002352961 |
| 154 | GO:0051707 | BP | response to other organism | 154/1349 | 2043/26994 | 4.05E-06 | 0.002352961 |
| 147 | GO:0098542 | BP | defense response to other organism | 147/1349 | 1906/26994 | 4.21E-06 | 0.002352961 |
| 154 | GO:0043207 | BP | response to external biotic stimulus | 154/1349 | 2069/26994 | 4.55E-06 | 0.002352961 |
| 159 | GO:0009607 | BP | response to biotic stimulus | 159/1349 | 2129/26994 | 4.70E-06 | 0.002352961 |
| 154 | GO:0044419 | BP | biological process involved in interspecies interaction between organisms | 154/1349 | 2044/26994 | 5.13E-06 | 0.002352961 |
| 260 | GO:0006950 | BP | response to stress | 260/1349 | 3782/26994 | 5.17E-06 | 0.002352961 |
| 298 | GO:0050896 | BP | response to stimulus | 298/1349 | 4429/26994 | 5.82E-06 | 0.002352961 |
| 168 | GO:0043531 | MF | ADP binding | 168/1349 | 1982/26994 | 3.74E-06 | 0.002352961 |
| 159 | GO:0009605 | BP | response to external stimulus | 159/1349 | 2208/26994 | 6.73E-06 | 0.002367604 |
| 17 | GO:0031225 | CC | anchored component of membrane | 17/1349 | 104/26994 | 1.52E-05 | 0.004390014 |
| 33 | GO:0022402 | BP | cell cycle process | 33/1349 | 294/26994 | 1.74E-05 | 0.004698606 |
| 6 | GO:0000727 | BP | double-strand break repair via break-induced replication | 6/1349 | 13/26994 | 1.95E-05 | 0.005092577 |
| 150 | GO:0007165 | BP | signal transduction | 150/1349 | 2149/26994 | 2.76E-05 | 0.006763515 |
| 5 | GO:0042555 | CC | MCM complex | 5/1349 | 9/26994 | 3.29E-05 | 0.007612513 |
| 7 | GO:0009522 | CC | photosystem I | 7/1349 | 21/26994 | 4.80E-05 | 0.010493852 |
| 21 | GO:0007049 | BP | cell cycle | 21/1349 | 168/26994 | 0.00010322 | 0.020372957 |
| 5 | GO:0009765 | BP | photosynthesis, light harvesting | 5/1349 | 11/26994 | 0.00011097 | 0.02095058 |
| 20 | GO:1903047 | BP | mitotic cell cycle process | 20/1349 | 161/26994 | 0.00016264 | 0.029246096 |
| 6 | GO:0016725 | MF | oxidoreductase activity, acting on CH or CH2 groups | 6/1349 | 18/26994 | 0.00017004 | 0.029275814 |
| 9 | GO:0044770 | BP | cell cycle phase transition | 9/1349 | 43/26994 | 0.00022867 | 0.034266369 |
| 9 | GO:0044772 | BP | mitotic cell cycle phase transition | 9/1349 | 43/26994 | 0.00022867 | 0.034266369 |
| 5 | GO:0000347 | CC | THO complex | 5/1349 | 13/26994 | 0.0002842 | 0.041067351 |

**Table S7.** KEGG pathways enrichment analysis

| Num | Pathway id | Description | Ratio_in_study | Ratio_in_pop | Pvalue_uncorrected | Pvalue_corrected |
| --- | --- | --- | --- | --- | --- | --- |
| 18 | map00073 | Cutin, suberine and wax biosynthesis | 18/593 | 106/11545 | 6.69E-06 | 0.000655543 |
| 31 | map04141 | Protein processing in endoplasmic reticulum | 31/593 | 280/11545 | 4.45E-05 | 0.002182056 |
| 5 | map00196 | Photosynthesis - antenna proteins | 5/593 | 11/11545 | 0.00012539 | 0.004096119 |
| 45 | map04626 | Plant-pathogen interaction | 45/593 | 511/11545 | 0.0002697 | 0.006607597 |
| 15 | map03030 | DNA replication | 15/593 | 125/11545 | 0.00183844 | 0.030027805 |
| 13 | map00040 | Pentose and glucuronate interconversions | 13/593 | 99/11545 | 0.00160777 | 0.031512386 |
| 11 | map00900 | Terpenoid backbone biosynthesis | 11/593 | 82/11545 | 0.00303004 | 0.042420582 |
| 12 | map00999 | Biosynthesis of various plant secondary metabolites | 12/593 | 112/11545 | 0.0122623 | 0.150213206 |
| 18 | map00940 | Phenylpropanoid biosynthesis | 18/593 | 200/11545 | 0.01469203 | 0.159979878 |
| 5 | map00908 | Zeatin biosynthesis | 5/593 | 38/11545 | 0.04350752 | 0.426373686 |
| 11 | map00480 | Glutathione metabolism | 11/593 | 129/11545 | 0.06771904 | 0.510497369 |
| 7 | map00380 | Tryptophan metabolism | 7/593 | 72/11545 | 0.0756688 | 0.529681618 |
| 2 | map00902 | Monoterpenoid biosynthesis | 2/593 | 8/11545 | 0.06002041 | 0.534727287 |
| 6 | map00195 | Photosynthesis | 6/593 | 56/11545 | 0.06631381 | 0.541562806 |
| 10 | map00270 | Cysteine and methionine metabolism | 10/593 | 123/11545 | 0.10072373 | 0.616932857 |
| 18 | map04075 | Plant hormone signal transduction | 18/593 | 251/11545 | 0.09558012 | 0.624456789 |
| 2 | map00590 | Arachidonic acid metabolism | 2/593 | 12/11545 | 0.12370964 | 0.673530275 |
| 4 | map00910 | Nitrogen metabolism | 4/593 | 37/11545 | 0.12000868 | 0.691814733 |
| 3 | map01040 | Biosynthesis of unsaturated fatty acids | 3/593 | 31/11545 | 0.21160506 | 0.829491821 |
| 8 | map00592 | alpha-Linolenic acid metabolism | 8/593 | 110/11545 | 0.20388756 | 0.832540878 |
| 5 | map00061 | Fatty acid biosynthesis | 5/593 | 61/11545 | 0.20306848 | 0.865248323 |
| 9 | map00941 | Flavonoid biosynthesis | 9/593 | 124/11545 | 0.1870401 | 0.8728538 |
| 5 | map00051 | Fructose and mannose metabolism | 5/593 | 65/11545 | 0.24081552 | 0.874071145 |
| 3 | map00945 | Stilbenoid, diarylheptanoid and gingerol biosynthesis | 3/593 | 30/11545 | 0.198129 | 0.88257462 |
| 7 | map00053 | Ascorbate and aldarate metabolism | 7/593 | 89/11545 | 0.17242944 | 0.889372904 |
| 10 | map00520 | Amino sugar and nucleotide sugar metabolism | 10/593 | 149/11545 | 0.23604311 | 0.889700964 |
| 3 | map00450 | Selenocompound metabolism | 3/593 | 29/11545 | 0.18487209 | 0.905873237 |
| 3 | map00906 | Carotenoid biosynthesis | 3/593 | 35/11545 | 0.26715417 | 0.935039597 |
| 3 | map00780 | Biotin metabolism | 3/593 | 35/11545 | 0.26715417 | 0.935039597 |
| 7 | map04146 | Peroxisome | 7/593 | 105/11545 | 0.29424026 | 0.961184849 |
| 1 | map00562 | Inositol phosphate metabolism | 1/593 | 78/11545 | 0.98387068 | 0.994013678 |
| 1 | map03018 | RNA degradation | 1/593 | 116/11545 | 0.99786272 | 0.997862715 |
| 3 | map00591 | Linoleic acid metabolism | 3/593 | 40/11545 | 0.33842481 | 1 |
| 1 | map00470 | D-Amino acid metabolism | 1/593 | 8/11545 | 0.34424905 | 1 |
| 2 | map00904 | Diterpenoid biosynthesis | 2/593 | 25/11545 | 0.37025801 | 1 |
| 5 | map01250 | Biosynthesis of nucleotide sugars | 5/593 | 78/11545 | 0.37237226 | 1 |
| 1 | map00909 | Sesquiterpenoid and triterpenoid biosynthesis | 1/593 | 9/11545 | 0.37795455 | 1 |
| 5 | map01232 | Nucleotide metabolism | 5/593 | 80/11545 | 0.39303896 | 1 |
| 4 | map00240 | Pyrimidine metabolism | 4/593 | 63/11545 | 0.40693946 | 1 |
| 5 | map00710 | Carbon fixation in photosynthetic organisms | 5/593 | 82/11545 | 0.41364991 | 1 |
| 12 | map00500 | Starch and sucrose metabolism | 12/593 | 214/11545 | 0.41964656 | 1 |
| 3 | map00565 | Ether lipid metabolism | 3/593 | 46/11545 | 0.42319098 | 1 |
| 13 | map04016 | MAPK signaling pathway - plant | 13/593 | 238/11545 | 0.4505818 | 1 |
| 4 | map00250 | Alanine, aspartate and glutamate metabolism | 4/593 | 67/11545 | 0.45344329 | 1 |
| 4 | map00052 | Galactose metabolism | 4/593 | 68/11545 | 0.46489185 | 1 |
| 5 | map00071 | Fatty acid degradation | 5/593 | 88/11545 | 0.47463614 | 1 |
| 10 | map04144 | Endocytosis | 10/593 | 188/11545 | 0.50131042 | 1 |
| 3 | map00280 | Valine, leucine and isoleucine degradation | 3/593 | 52/11545 | 0.50395154 | 1 |
| 4 | map00400 | Phenylalanine, tyrosine and tryptophan biosynthesis | 4/593 | 74/11545 | 0.53154265 | 1 |
| 3 | map00130 | Ubiquinone and other terpenoid-quinone biosynthesis | 3/593 | 55/11545 | 0.54209577 | 1 |
| 2 | map00790 | Folate biosynthesis | 2/593 | 35/11545 | 0.54310738 | 1 |
| 2 | map00650 | Butanoate metabolism | 2/593 | 35/11545 | 0.54310738 | 1 |
| 2 | map00062 | Fatty acid elongation | 2/593 | 37/11545 | 0.57353211 | 1 |
| 2 | map00310 | Lysine degradation | 2/593 | 38/11545 | 0.58818208 | 1 |
| 4 | map00460 | Cyanoamino acid metabolism | 4/593 | 82/11545 | 0.61348173 | 1 |
| 4 | map04145 | Phagosome | 4/593 | 82/11545 | 0.61348173 | 1 |
| 1 | map00740 | Riboflavin metabolism | 1/593 | 19/11545 | 0.63310501 | 1 |
| 4 | map00350 | Tyrosine metabolism | 4/593 | 86/11545 | 0.65095679 | 1 |
| 2 | map00360 | Phenylalanine metabolism | 2/593 | 43/11545 | 0.65579394 | 1 |
| 4 | map00230 | Purine metabolism | 4/593 | 87/11545 | 0.6599381 | 1 |
| 1 | map00730 | Thiamine metabolism | 1/593 | 21/11545 | 0.66988808 | 1 |
| 1 | map00950 | Isoquinoline alkaloid biosynthesis | 1/593 | 21/11545 | 0.66988808 | 1 |
| 3 | map00630 | Glyoxylate and dicarboxylate metabolism | 3/593 | 67/11545 | 0.67616634 | 1 |
| 1 | map00511 | Other glycan degradation | 1/593 | 22/11545 | 0.68687492 | 1 |
| 2 | map03430 | Mismatch repair | 2/593 | 46/11545 | 0.69192917 | 1 |
| 1 | map00430 | Taurine and hypotaurine metabolism | 1/593 | 24/11545 | 0.71827525 | 1 |
| 2 | map00600 | Sphingolipid metabolism | 2/593 | 49/11545 | 0.72487903 | 1 |
| 3 | map03020 | RNA polymerase | 3/593 | 73/11545 | 0.73138014 | 1 |
| 2 | map03410 | Base excision repair | 2/593 | 51/11545 | 0.74515241 | 1 |
| 1 | map00670 | One carbon pool by folate | 1/593 | 26/11545 | 0.74653151 | 1 |
| 3 | map03440 | Homologous recombination | 3/593 | 77/11545 | 0.763894 | 1 |
| 3 | map03420 | Nucleotide excision repair | 3/593 | 79/11545 | 0.77891455 | 1 |
| 4 | map00564 | Glycerophospholipid metabolism | 4/593 | 105/11545 | 0.79465477 | 1 |
| 5 | map00190 | Oxidative phosphorylation | 5/593 | 130/11545 | 0.80437775 | 1 |
| 2 | map04712 | Circadian rhythm - plant | 2/593 | 58/11545 | 0.80628735 | 1 |
| 1 | map00920 | Sulfur metabolism | 1/593 | 32/11545 | 0.81542547 | 1 |
| 1 | map04130 | SNARE interactions in vesicular transport | 1/593 | 34/11545 | 0.83395034 | 1 |
| 1 | map00220 | Arginine biosynthesis | 1/593 | 34/11545 | 0.83395034 | 1 |
| 1 | map00944 | Flavone and flavonol biosynthesis | 1/593 | 34/11545 | 0.83395034 | 1 |
| 1 | map03060 | Protein export | 1/593 | 39/11545 | 0.87254183 | 1 |
| 6 | map00010 | Glycolysis / Gluconeogenesis | 6/593 | 171/11545 | 0.87892522 | 1 |
| 4 | map00620 | Pyruvate metabolism | 4/593 | 125/11545 | 0.89024702 | 1 |
| 1 | map03250 | Viral life cycle - HIV-1 | 1/593 | 44/11545 | 0.90217585 | 1 |
| 1 | map00960 | Tropane, piperidine and pyridine alkaloid biosynthesis | 1/593 | 47/11545 | 0.9165419 | 1 |
| 1 | map00020 | Citrate cycle (TCA cycle) | 1/593 | 49/11545 | 0.92492883 | 1 |
| 7 | map03040 | Spliceosome | 7/593 | 221/11545 | 0.94125865 | 1 |
| 2 | map03008 | Ribosome biogenesis in eukaryotes | 2/593 | 87/11545 | 0.94252107 | 1 |
| 1 | map00260 | Glycine, serine and threonine metabolism | 1/593 | 55/11545 | 0.94536916 | 1 |
| 4 | map02010 | ABC transporters | 4/593 | 150/11545 | 0.953486 | 1 |
| 1 | map00030 | Pentose phosphate pathway | 1/593 | 61/11545 | 0.96025078 | 1 |
| 8 | map03010 | Ribosome | 8/593 | 260/11545 | 0.96066539 | 1 |
| 6 | map04120 | Ubiquitin mediated proteolysis | 6/593 | 214/11545 | 0.96691924 | 1 |
| 1 | map00860 | Porphyrin metabolism | 1/593 | 65/11545 | 0.96784752 | 1 |
| 1 | map00410 | beta-Alanine metabolism | 1/593 | 65/11545 | 0.96784752 | 1 |
| 9 | map01240 | Biosynthesis of cofactors | 9/593 | 294/11545 | 0.96940486 | 1 |
| 1 | map00330 | Arginine and proline metabolism | 1/593 | 66/11545 | 0.96950836 | 1 |
| 3 | map03013 | Nucleocytoplasmic transport | 3/593 | 137/11545 | 0.97456857 | 1 |
| 1 | map00561 | Glycerolipid metabolism | 1/593 | 76/11545 | 0.98206379 | 1 |

**
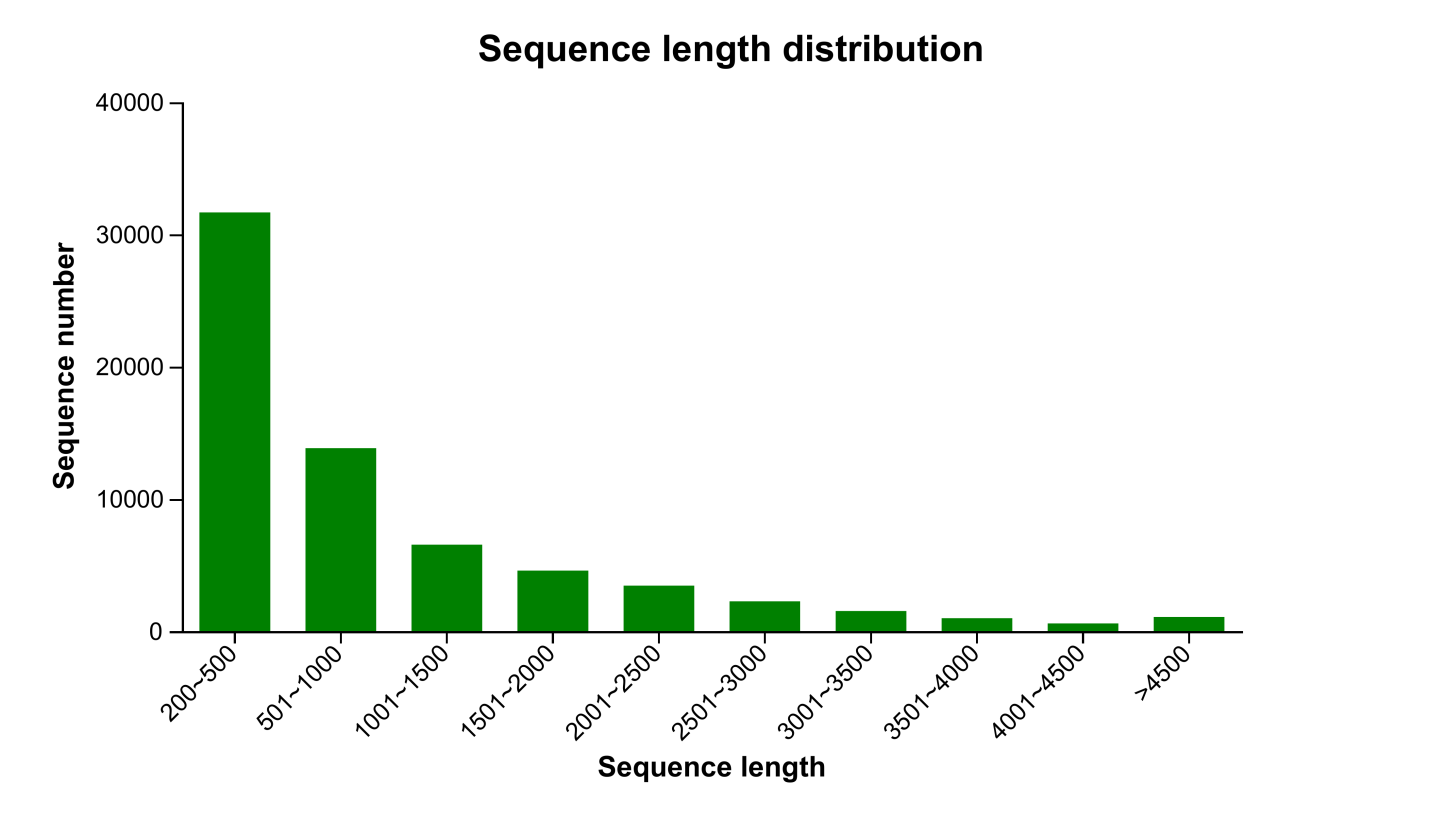
**

**Fig. S1.** Sequence length distribution. Abscissa: length range of unigene; ordinate: number of unigenes within this length range.

**
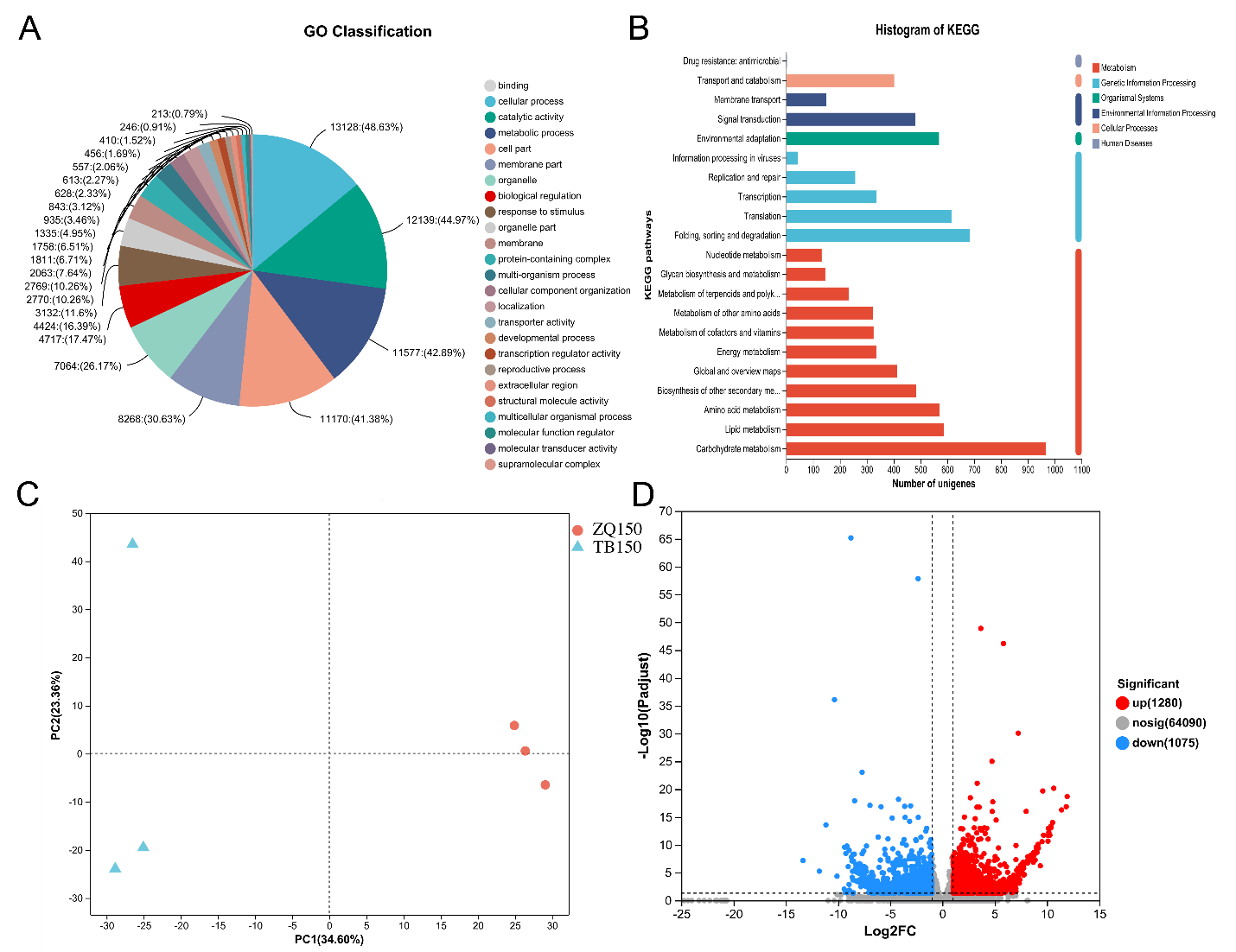
**

**Fig. S2.** Functional annotation of generated unigenes. Unigenes were annotated with gene ontology (GO) analysis; the different colors of each pie chart represent different GO terms, and its area indicates the relative proportion of unigenes in the GO term (A). Unigenes annotated with Kyoto Encyclopedia of Genes and Genomes (KEGG) analysis: the y-axis represents the name of the KEGG metabolic pathway, and the x-axis represents the number of unigenes annotated to the pathway (B). Principal component analysis (PCA) represents the distance between both samples (C). The volcano indicates the differentially expressed genes (DEGs). Each dot in the figure signifies a particular DEG. The red dot shows upregulated unigenes, the blue dots indicate downregulated unigenes, and the gray dots show the nonsignificant differential unigenes (D).

**
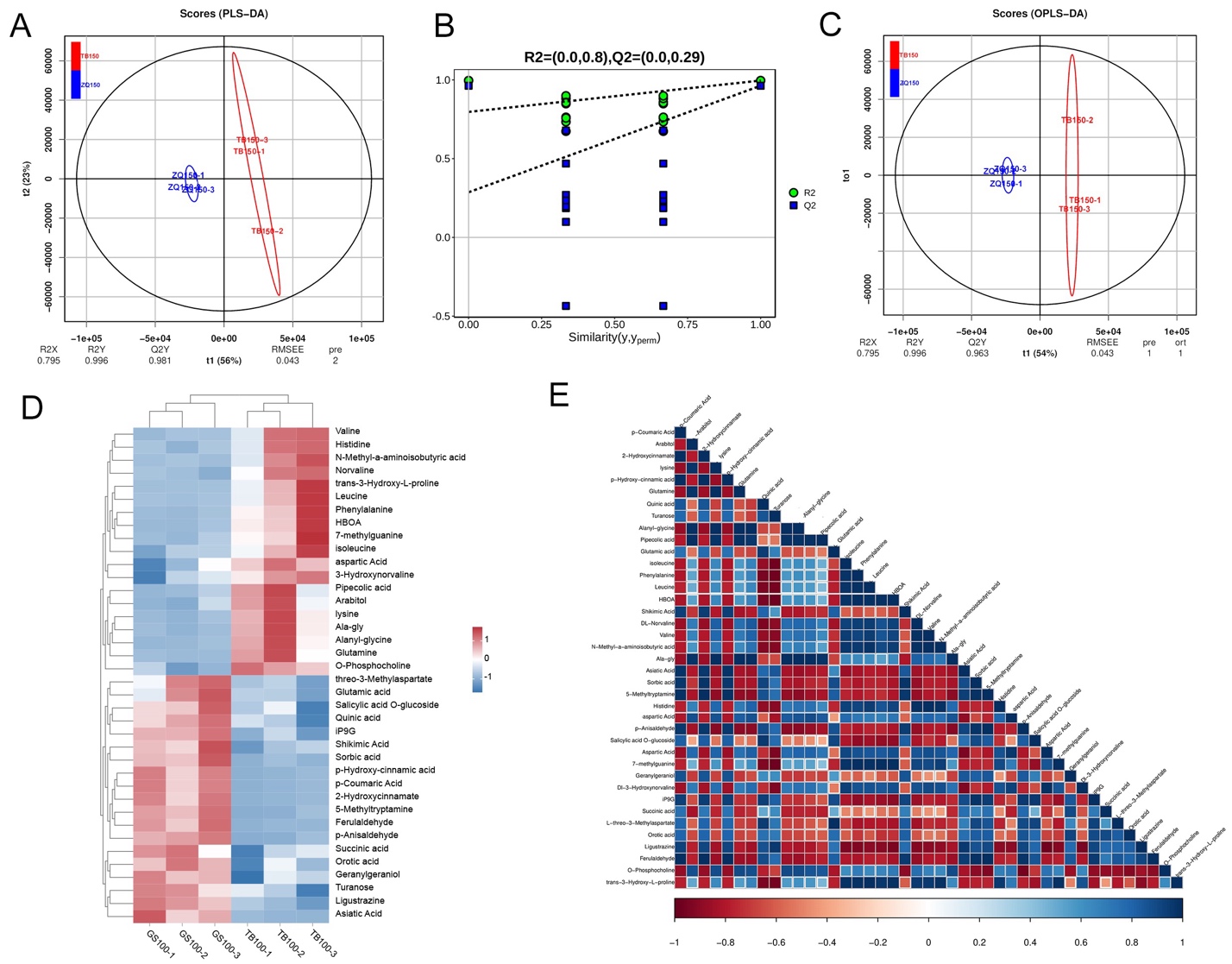
**

**Fig. S3.** Metabolomics analysis. Orthogonal Partial Least Squares Discriminant Analysis (OPLS-DA) (A)**.** OPLS-DA permutation test chart (B). Partial Least Squares Discriminant Analysis (PLS-DA) (C)**.** Differential metabolite clustering heatmap (D). Differential metabolite correlation heatmap (E).

**
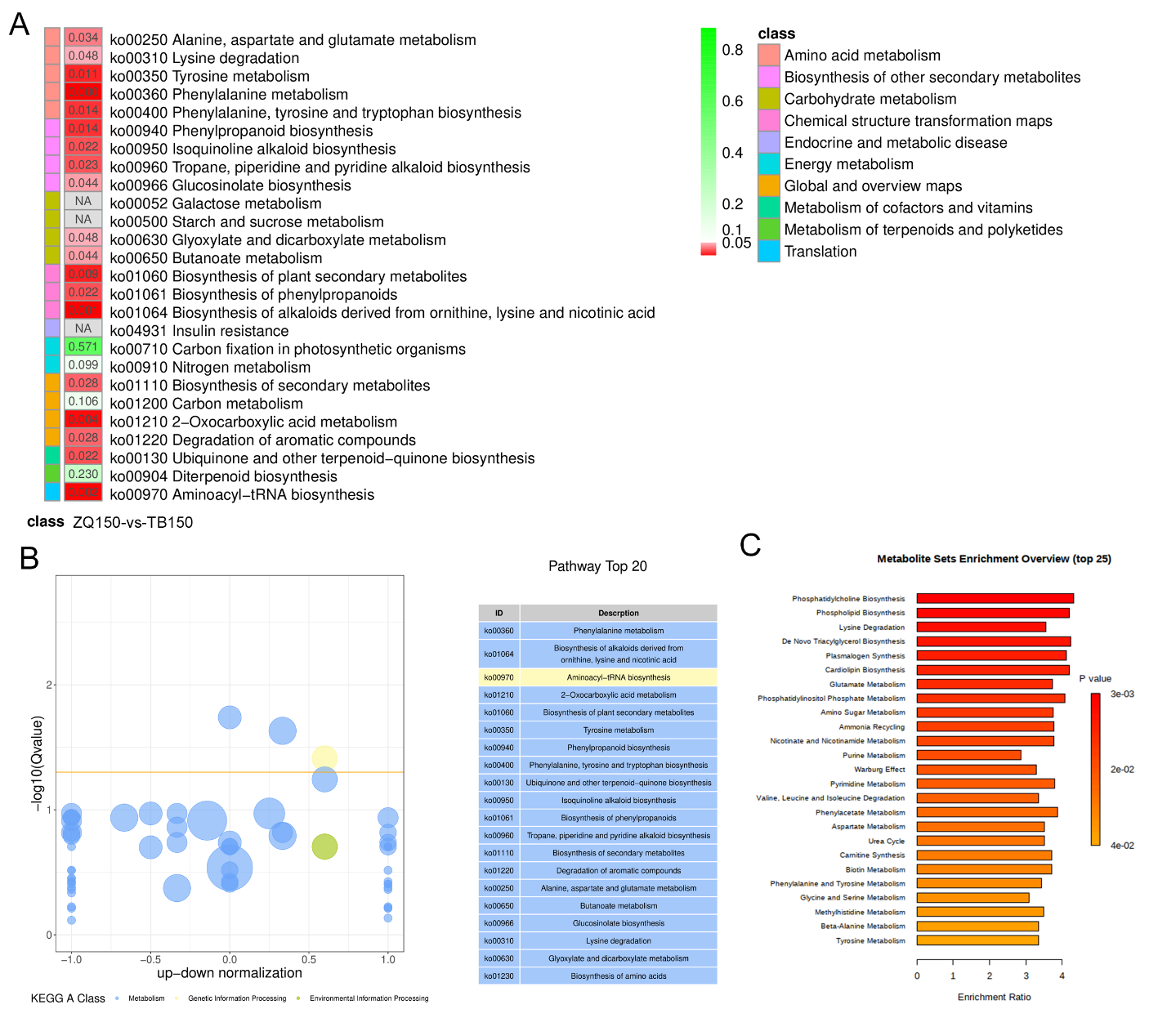
**

**Fig. S4.** Metabolic pathway analysis. Kyoto Encyclopedia of Genes and Genomes (KEGG) pathway assignment of all metabolites among TB150 vs ZQ150 (A). KEGG pathway assignment of differential metabolites among TB150 vs ZQ150 (B). MSEA enrichment map (C).

**
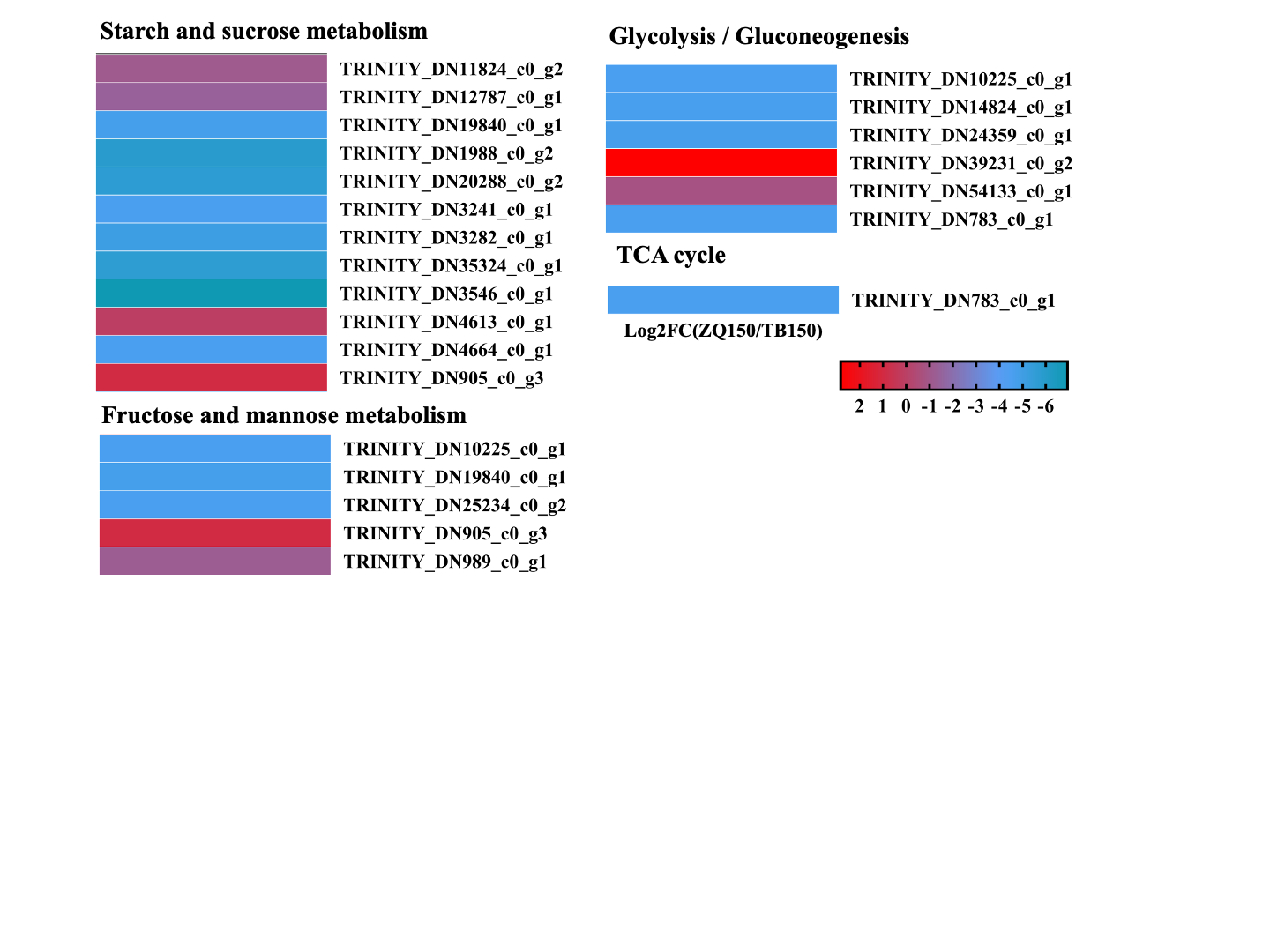
**

**Fig. S5.** Selected differentially expressed genes (DEGs) related to sugar metabolism were identified from RNA sequencing data. Heat map diagram of the log2FC; the color scale represents the expression levels from lower to higher.
